# *In vitro* Characterization of Phenylacetate Decarboxylase, a Novel Enzyme Catalyzing Toluene Biosynthesis in an Anaerobic Microbial Community

**DOI:** 10.1038/srep31362

**Published:** 2016-08-10

**Authors:** K. Zargar, R. Saville, R. M. Phelan, S. G. Tringe, C. J. Petzold, J. D. Keasling, H. R. Beller

**Affiliations:** 1 Joint BioEnergy Institute (JBEI), 5885 Hollis Avenue, Emeryville, CA, USA; 2 QB3 Institute, University of California, Berkeley, California 94270, United States; 3 Joint Genome Institute, 2800 Mitchell Drive, Walnut Creek, CA, USA; 4 Biosciences, Lawrence Berkeley National Laboratory (LBNL), Berkeley, CA, USA; 5 Departments of Chemical & Biomolecular Engineering and of Bioengineering, University of California, Berkeley, CA, USA; 6 Novo Nordisk Foundation Center for Biosustainability, Technical University of Denmark, Kogle Allé, DK2970-Hørsholm, Denmark; 7 Earth and Environmental Sciences, LBNL, Berkeley, CA, USA

## Abstract

Anaerobic bacterial biosynthesis of toluene from phenylacetate was reported more than two decades ago, but the biochemistry underlying this novel metabolism has never been elucidated. Here we report results of *in vitro* characterization studies of a novel phenylacetate decarboxylase from an anaerobic, sewage-derived enrichment culture that quantitatively produces toluene from phenylacetate; complementary metagenomic and metaproteomic analyses are also presented. Among the noteworthy findings is that this enzyme is not the well-characterized clostridial *p*-hydroxyphenylacetate decarboxylase (CsdBC). However, the toluene synthase under study appears to be able to catalyze both phenylacetate and *p*-hydroxyphenylacetate decarboxylation. Observations suggesting that phenylacetate and *p*-hydroxyphenylacetate decarboxylation in complex cell-free extracts were catalyzed by the same enzyme include the following: (i) the specific activity for both substrates was comparable in cell-free extracts, (ii) the two activities displayed identical behavior during chromatographic separation of cell-free extracts, (iii) both activities were irreversibly inactivated upon exposure to O_2_, and (iv) both activities were similarly inhibited by an amide analog of *p*-hydroxyphenylacetate. Based upon these and other data, we hypothesize that the toluene synthase reaction involves a glycyl radical decarboxylase. This first-time study of the phenylacetate decarboxylase reaction constitutes an important step in understanding and ultimately harnessing it for making bio-based toluene.

Toluene is an important petrochemical with a global market of 29 million tons per year^1^ whose uses include synthesis of other aromatic feedstocks and serving as an effective octane booster in gasoline (octane number, 114). From the perspective of global sustainability, it would be desirable to offset the enormous volume of petroleum-derived toluene with biochemically produced toluene made from a renewable resource, such as lignocellulosic biomass. Engineering microbes to produce toluene from cellulosic sugars would require knowledge of enzymes capable of its biosynthesis. In fact, microbial toluene biosynthesis has been reported in a variety of anaerobic environments ranging from anoxic lake sediments to sewage treatment facilities^2^,^3^,^4^. For example, bacterial toluene biosynthesis was reported in the gamma proteobacterium *Tolumonas auensis*, which was isolated from anaerobic lake sediments^5^. *T. auensis* was observed to generate toluene from phenylalanine and several other aromatic compounds, namely, phenyllactate, phenylpyruvate, and phenylacetate^5^. The first three compounds listed are all plausible precursors of phenylacetate (e.g., phenylalanine and phenyllactate can be converted to phenylpyruvate by transamination and dehydrogenase reactions, respectively, and phenylpyruvate can be converted to phenylacetate by decarboxylase and dehydrogenase reactions). Thus, phenylacetate is arguably the direct substrate for the toluene synthase in *T. auensis*. Another bacterium, *Clostridium aerofoetidum* strain WS, was also reported to make toluene from phenylacetate or phenylalanine^6^. However, the underlying biochemistry and genetics of the conversion of phenylacetate to toluene have never been elucidated in any organism.

The known enzyme with the greatest functional similarity to the as-yet unidentified enzyme that converts phenylacetate to toluene is *p*-hydroxyphenylacetate decarboxylase, also known as HpdBC (in *Peptoclostridium difficile*, formerly *Clostridium difficile*) or CsdBC (in *Clostridium scatalogenes*), which converts *p*-hydroxyphenylacetate to *p*-cresol^7^,^8^,^9^. Thus, the substrates and products for these reactions differ only by a *para*-hydroxy group. *p*-Hydroxyphenylacetate decarboxylase is a glycyl radical enzyme that has been structurally characterized and whose Kolbe-type decarboxylation mechanism has been described^10^,^11^. The *para*-hydroxy group is proposed to play an essential role in the *p*-hydroxyphenylacetate decarboxylase reaction: in an unusual mechanism proposed for *p*-hydroxyphenylacetate activation, there is a concerted abstraction of a proton from the *para*-hydroxy group by Glu637 and abstraction of an electron from the carboxyl group by Cys503^11^. Together, the proton and electron abstraction constitute a *de facto* H atom abstraction, although the abstraction occurs in two distinct locations on the substrate molecule. Initial studies of HpdBC suggested that it cannot catalyze phenylacetate decarboxylation^7^.

In this paper, we describe development of an anaerobic, toluene-producing enrichment culture and initial biochemical characterization of the enzyme activity in that culture that catalyzes conversion of phenylacetate to toluene. We present *in vivo* studies and genome-informed *in vitro* biochemical studies of cell-free and partially purified extracts addressing reaction stoichiometry, enzyme solubility and sensitivity to O_2_, substrate range and enzyme inhibition in the presence of phenylacetate analogs, and similarities and differences with respect to the well characterized potential homolog, *p*-hydroxyphenylacetate decarboxylase. Based upon these data, we speculate on the nature of the novel toluene synthase reaction, in particular, distinguishing between phenylacetate decarboxylation *versus* phenylacetate reduction and phenylacetaldehyde decarbonylation.

## Results

### *Tolumonas auensis* and *Clostridium aerofoetidum* not observed to make toluene under previously reported conditions

Despite numerous attempts, we were unable to replicate the reported anaerobic or aerobic biosynthesis of toluene from phenylacetate by *Tolumonas auensis* strain TA 4 (DSM 9187) under the published conditions^5^. Although our work was nominally performed with the same strain as that used by Fischer-Romero *et al*.^5^, there is some sequence divergence (~99.15% identity) between the rRNA gene sequence reported by Fischer-Romero *et al*. and that in the strain that we studied and sequenced^12^. We were also unable to replicate anaerobic toluene biosynthesis from phenylacetate by *C. aerofoetidum* under reported conditions^6^; there is some uncertainty about whether the strain used for the original 1984 report was exactly the same as the one currently available from culture collections (see Materials and Methods).

### Establishment and community (including metagenomic) characterization of a toluene-producing enrichment culture inoculated with sewage sludge

As we were unable to replicate toluene biosynthesis in *T. auensis* or *C. aerofoetidum*, we attempted to establish a toluene-producing culture from an inoculum source previously reported to harbor such activity^4^. Inoculation of anaerobic, modified TP growth medium (see Materials and Methods) with sludge from a municipal wastewater treatment plant and cultivation under anaerobic conditions in a glove box resulted in toluene biosynthesis from phenylacetate (confirmed in cell suspension experiments with ^13^C-labeled compounds, as described below). Community analysis with 16S rRNA gene iTags showed that the community was dominated by an OTU (Operational Taxonomic Unit) representing one or more members of the *Enterobacteriaceae* family (~67% relative abundance), followed in abundance by OTUs representing members of the genera *Acidaminococcus* (7.5%), *Desulfovibrio* (3.9%), *Methanobacterium* (3.6%), *Kluyvera* (3.0%), and others with relative abundances of less than 3%. No OTUs representing *Tolumonas* were detected. Detailed results of this analysis are presented in Supplementary Table S1.

Although the toluene-producing microbial community was dominated by members of the *Enterobacteriaceae* family, the metagenome (JGI IMG Taxon ID 3300001784) included a large number (343,121) of putative protein-coding genes, equivalent to the genomes of more than 50 bacteria/archaea. 4420 of these genes had > 90% translated sequence identity to proteins belonging to sequenced *Enterobacter* isolates in GenBank. Notably, the metagenome encoded two apparent copies of *p*-hydroxyphenylacetate decarboxylase (CsdBC), a possible homolog for a phenylacetate decarboxylase that could produce toluene. The two copies of *csdBCA* (where *csdA* encodes the cognate glycyl radical activating enzyme) identified in the metagenome were JGI20225J20221_10013067 to 65 (*csdBCA*) and JGI20225J20221_100001556 to 58 (*csdBCA*). The translated versions of these *csdB* copies had 67–73% sequence identity with the version from *C. scatologenes*, had the expected active site residues described by Martins *et al*.^10^ (e.g., Arg223, Ser344, Gly345, Phe405, Cys503, Glu505, Gly532, His536, Glu637, using *C. scatologenes* numbering), and had gene synteny of *csdBCA* identical to that observed in *C. scatologenes*^8^. This finding is discussed further below with respect to shotgun proteomic analyses of toluene-producing fractions of the cell-free extract after partial purification (the primary purpose of the metagenome sequence in this study was to provide a database for mapping peptides from shotgun proteomics).

### Stoichiometric conversion of phenylacetate (and phenylalanine) to toluene by enrichment cultures

**Figure 1:**
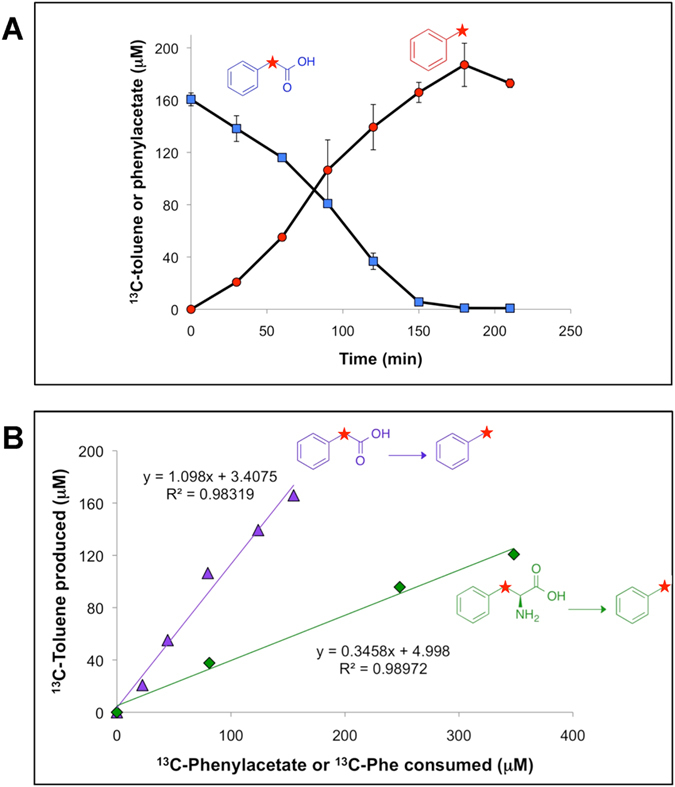
Conversion of ^13^C-phenylacetate and ^13^C-phenylalanine (^13^C-Phe) to ^13^C-toluene catalyzed by anaerobic cell suspensions of a sewage-derived enrichment culture. (**A**) Quantitative conversion of phenylacetate to toluene, (**B**) stoichiometry of phenylacetate and phenylalanine conversion to toluene (linear regression fits are shown). Error bars represent one standard deviation. Red stars on chemical structures represent ^13^C atoms.

Enrichment cultures derived from the sewage sludge inoculum quantitatively converted phenylacetate to toluene. For example, results of a cell suspension experiment demonstrating quantitative production of [*methyl*-^13^C]toluene from 200 μM phenylacetic acid-2-^13^C are shown in Fig. 1A. Linear regression (Fig. 1B) revealed that the ratio of toluene produced/phenylacetate consumed was approximately 1 (1.1; *r*^2^ = 0.98), or 100 mol%, indicating quantitative conversion.

For toluene-producing microbial communities in natural or engineered systems, such as anoxic lake sediment^2^,^3^ or wastewater treatment plants^4^, it is likely that the ultimate source of toluene is phenylacetate derived from L-phenylalanine degradation. Thus, we assessed whether toluene could be produced from phenylalanine in these enrichment cultures. One possible pathway for phenylalanine to phenylacetate entails transamination of phenylalanine to phenylpyruvate (e.g., *via* phenylalanine transaminase; EC 2.6.1.57), decarboxylation to phenylacetaldehyde (e.g., *via* phenylpyruvate decarboxylase; EC 4.1.1.43), and oxidation to phenylacetate (e.g., *via* phenylacetaldehyde dehydrogenase; EC 1.2.1.39), although other pathways are possible^13^. Cell suspension experiments with L-phenylalanine-β-^13^C showed stoichiometric conversion of the amino acid to [*methyl*-^13^C]toluene (Fig. 1B). Phenylalanine consumption was highly correlated to toluene production (*r*^2^ = 0.99) with ca. 35 mol% conversion. These results are consistent with L-phenylalanine being the ultimate source of toluene in the cultures when they are not amended directly with phenylacetate. The relatively low (35 mol%) yield of toluene from phenylalanine could be explained by diversion of a fraction (65 mol%) of intermediates from phenylalanine conversion to phenylacetate (e.g., phenylpyruvate, phenylacetaldehyde) toward metabolic pathways that do not result in phenylacetate formation. Based on the results presented in Fig. 1, it appears that any phenylacetate derived from phenylalanine would be quantitatively converted to toluene, even in this complex microbial community.

### *In vitro* studies show that the phenylacetate decarboxylase is soluble

*In vitro* assays measuring conversion of phenylacetic acid-2-^13^C to [*methyl*-^13^C]toluene were conducted on crude extract from the enrichment culture and the supernatant and pellet after the extract was spun in an ultracentrifuge at 150,000×*g* for 60 min at 4 °C. Cells were broken by passage through a French pressure cell (three times) under anaerobic conditions and assays were performed under anaerobic conditions in a glove box. The supernatant accounted for 63.8% of the crude extract activity, whereas the resuspended pellet accounted for 31.6% of the crude extract activity. Thus, toluene synthase in the enrichment cultures can be considered to be a soluble enzyme.

### *In vitro* studies show that the phenylacetate decarboxylase activity is not diminished by dialysis

Cell-free extracts from the enrichment cultures were dialyzed to examine requirements for diffusible cofactors. Aliquots of extract dialyzed for 8 hr using a 3.5-kDa molecular weight cutoff membrane did not have decreased phenylacetate decarboxylase activity relative to an undialyzed control; indeed, they had greater activity than the undialyzed control (possibly due to removal of inhibitors during dialysis)(Supplementary Fig. S1). Dialysis was shown to be effective by independent trials that contained extract amended with 10 mM sodium bromide tracer; these trials demonstrated that 99% of the bromide was removed within 4 hr of dialysis (well within the total dialysis treatment time of 8 hr).

### Partial purification of phenylacetate decarboxylase and proteomic analysis of active fractions do not indicate a role for the known *p*-hydroxyphenylacetate decarboxylase (CsdBC)

**Figure 2:**
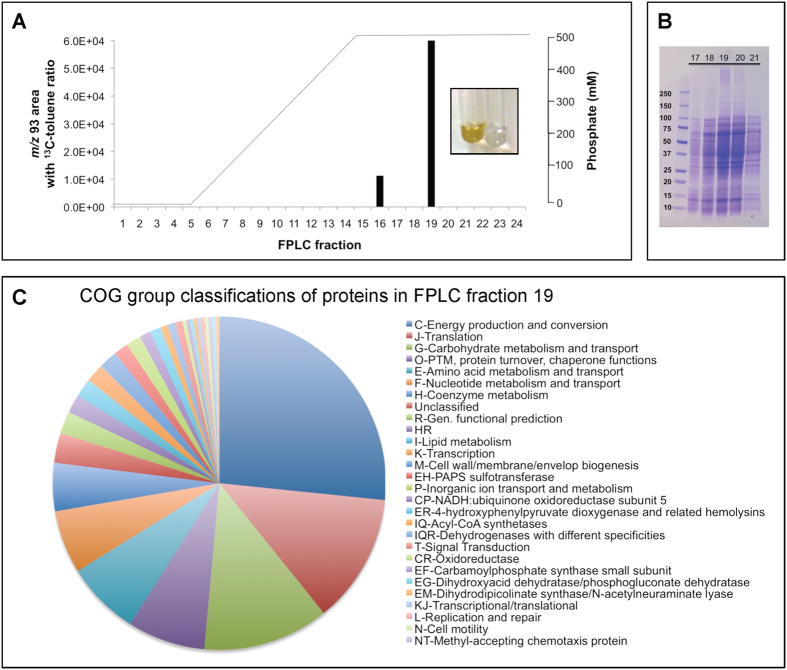
Results from partial purification of phenylacetate decarboxylase activity from a sewage-derived enrichment culture using a hydroxyapatite column. (**A**) Activity as indicated by *m/z* 93 area of a GC/MS peak with an *m/z* 93/92 ratio (0.60 to 0.62) consistent with [*methyl*-^13^C]toluene (see the Supplementary Material for details on calculations of *m/z* 92 and 93 areas); the phosphate gradient used for elution is also shown, along with the brown-yellow color of the most active fraction compared with assay buffer, (**B**) SDS-PAGE gel of active and adjacent inactive fractions from an FPLC purification, and (**C**) COG assignments of proteins that are more abundant in the most active fraction (#19) than in an inactive fraction (#18).

In an attempt to narrow down the list of possible phenylacetate decarboxylase gene candidates, cell-free extracts of the enrichment culture were subjected to chromatographic separation with a hydroxyapatite column and the active fraction(s) were characterized by shotgun proteomics. Such a purification experiment is depicted in Fig. 2A, where 1-mL fractions from anaerobic FPLC (Fast Protein Liquid Chromatography) purification are represented on the *x*-axis and the *m/z* 93 area is shown on the *y*-axis for peaks that had a characteristic *m/z* 92/93 ratio for [*methyl*-^13^C]toluene (0.6 + 0.02). The phenylacetate decarboxylase activity in this experiment was concentrated in fraction #19; some activity was also apparent in fraction #16, however, the GC/MS signal for this fraction was relatively close to the detection limit and therefore is less reliable. Fraction #19 was also characterized by a yellow color (Fig. 2A); overall, we consistently observed that the fractions with the darkest yellow color had the greatest toluene synthase activity.

Even after separation by elution through a hydroxyapatite column, the protein composition of the fraction was still complex (Fig. 2B). Shotgun proteomics revealed that 453 proteins were detected in the active fraction overall, and that 278 of these proteins had a higher abundance (e.g., greater number of total unique peptides) in the active fraction than in the adjacent inactive fraction (#18). To provide some context, 278 proteins represents less than 0.1% of the more than 343,000 proteins encoded in the corresponding metagenome of the enrichment culture. A summary of these proteins with respect to putative functional annotation, represented as COG (Clusters of Orthologous Groups) categories, is displayed in Fig. 2C. The largest COG category represented was C, Energy Production and Conversion. A more detailed listing of these proteins and their annotations and relative abundance is presented in Supplementary Table S2.

Notably absent among the 278 proteins was *p*-hydroxyphenylacetate decarboxylase (CsdBC), two copies of which were identified in the metagenome, as mentioned earlier. While Csd proteins were not detected, another glycyl radical enzyme, pyruvate formate-lyase (PFL; 99% protein sequence identity to an *Enterobacter asburiae* version, GenBank accession WP_048979720.1), was identified, but it was relatively abundant in both the active and inactive fractions (#18 and 19) (Supplementary Table S2). The absence of CsdBC among the identified proteins is consistent with, but does not prove, that the toluene biosynthesis enzyme in the enrichment culture is not a known *p*-hydroxyphenylacetate decarboxylase.

### *In vitro* studies with *p*-hydroxyphenylacetate decarboxylase (CsdBC) from *Clostridium scatologenes* indicate that it is not a phenylacetate decarboxylase

As a further, more definitive test of whether phenylacetate decarboxylase in the enrichment culture is actually a homolog of known *p*-hydroxyphenylacetate decarboxylase, we conducted *in vitro* studies with *C. scatologenes*, which natively expresses this enzyme (CsdBC). CsdBC was chosen in part because two apparent orthologs of CsdBC were identified in the sewage culture metagenome (as discussed earlier). Notably, these orthologs included conserved and putatively essential residues reported to be specifically associated with the *para*-hydroxy moiety in *p*-hydroxyphenylacetate (in particular, Glu637)^10^,^11^, which would not be applicable to unsubstituted phenylacetate.

Cell-free anaerobic extracts were amended with (a) no substrate, (b) *p*-hydroxyphenylacetate (1 mM), (c) phenylacetic acid-2-^13^C (1 mM), and (d) a combination of *p*-hydroxyphenylacetate + phenylacetic acid-2-^13^C (1 mM each). The assay mixtures were incubated under anaerobic conditions and then analyzed by GC/MS for *p*-cresol (from *p*-hydroxyphenylacetate) and ^13^C-toluene (from ^13^C-phenylacetate). The results of the assays (Supplementary Fig. S2) showed that CsdBC in the extracts produced *p*-cresol but not ^13^C-toluene: assays with 1 mM *p*-hydroxyphenylacetate yielded more than 30 μM *p*-cresol, whereas ^13^C-toluene was not detectable ( < 0.1 μM detection limit) in any assays with 1 mM phenylacetate.

### *In vitro* studies with partially purified cell extracts suggest that the novel phenylacetate decarboxylase is also a novel *p*-hydroxyphenylacetate decarboxylase

**Figure 3:**
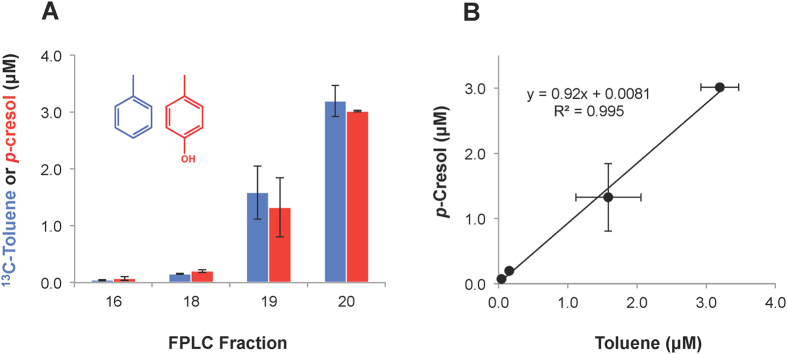
Toluene and *p*-cresol production from phenylacetate and *p*-hydroxyphenylacetate, respectively, in selected FPLC fractions during partial purification, as (**A**) a histogram, and (**B**) a scatterplot with a linear regression fit. Error bars represent one standard deviation. Note that both phenylacetate and *p*-hydroxyphenylacetate were added to the assay mixtures of each FPLC fraction shown, and were thus exposed to the exact same proteins for the same amount of time (24 hr). Thus, the ratio of final toluene and *p*-cresol concentrations (slope in panel **B**), is equivalent to the ratio of phenylacetate decarboxylase and *p*-hydroxyphenylacetate decarboxylase specific activities.

*In vitro* assays conducted with a series of FPLC fractions from hydroxyapatite chromatographic separation of cell-free enrichment culture extracts revealed an unexpected result: among these fractions, which spanned a range of phenylacetate decarboxylase activity, there was a very strong correlation between toluene and *p*-cresol production, suggesting that the same enzyme might be catalyzing decarboxylation of both phenylacetate and *p*-hydroxyphenylacetate with comparable specific activity. Figure 3A presents ^13^C-labeled toluene and *p*-cresol from *in vitro* assays of FPLC fractions amended with both *p*-hydroxyphenylacetate and ^13^C-labeled phenylacetate, and Fig. 3B shows a linear regression fit for these data. The correlation between toluene and *p*-cresol production is very strong (*r*^2^ = 0.995) and the slope is near unity (0.92), indicating either that the enzymes catalyzing phenylacetate and *p*-hydroxyphenylacetate carboxylation have near-identical chromatographic behavior and specific activity or, more likely, that the same enzyme is catalyzing both reactions with similar efficiency.

Comparison of Figs 2A and 3A shows that there was some variability in elution of toluene synthase activity in this low-resolution FPLC system. In particular, although fractions 19 and 20 consistently contained the vast majority of the observed activity, we observed variability in the relative distribution of activity between these two fractions. This variability has no effect on results reported here, as activity was directly measured, not assumed, in all experiments involving FPLC purification (i.e., Figs 2 and 3, and proteomics in Supplementary Table S2).

### Phenylacetate decarboxylase is irreversibly inactivated by O_2_

Cell-free extracts of the enrichment culture were tested for sensitivity to O_2_ of both phenylacetate and *p*-hydroxyphenylacetate decarboxylase activity. For both activities, < 5% of the activity remained after the cell-free extract was very slowly bubbled with air that was manually injected with an air-filled syringe for 5 min (Supplementary Fig. S3). Activity did not return after the extracts were re-reduced with 25 mM dithiothreitol. Irreversible inactivation by molecular oxygen is a characteristic associated with glycyl radical enzymes^14^,^15^.

### Phenylacetate decarboxylase activity with substrate analogs

**Figure 4:**
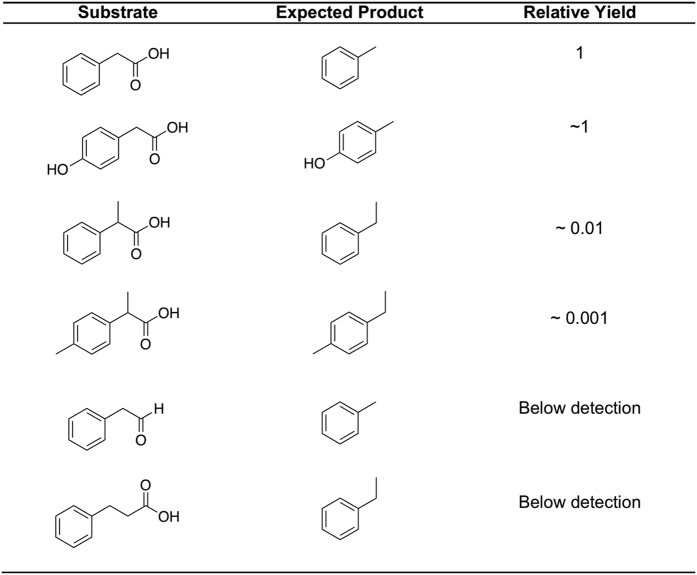
Phenylacetate decarboxylase activity on substrate analogs. Product yield shown is relative to toluene from phenylacetate (set as 1).

The ability of phenylacetate decarboxylase to act on various phenylacetate analogs was investigated with cell-free extracts of the enrichment culture (Fig. 4). As discussed previously, activity on *p*-hydroxyphenylacetate was comparable to that on phenylacetate. The presence of a methyl group on the methylene carbon of phenylacetate (2-phenylpropionate) resulted in a two order of magnitude decrease in decarboxylation activity, whereas an additional methyl group in the *para* position of the ring [2-(*p*-tolyl)propionic acid] resulted in an additional order of magnitude decrease in activity (Fig. 4). No decarboxylase activity was detected for 3-phenylpropionic acid and no decarbonylase activity was observed for phenylacetaldehyde.

### Phenylacetate decarboxylase is inhibited by certain amide substrate analogs

**Figure 5:**
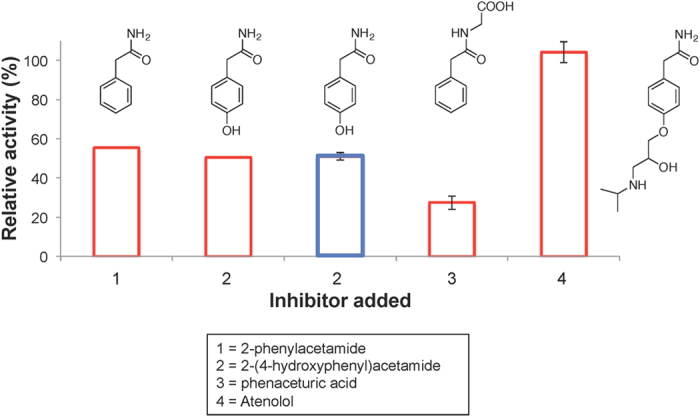
Phenylacetate decarboxylase activity in the presence of potential inhibitors, including 2-phenylacetamide, 2-(4-hydroxyphenyl)acetamide, phenaceturic acid, and Atenolol. For 2-(4-hydroxyphenyl)acetamide, inhibition of *p*-hydroxyphenylacetate decarboxylase activity is also shown (in blue). Activity is shown as relative to toluene production from phenylacetate in the absence of an inhibitor, except for the blue bar, which is relative to *p*-cresol production from *p*-hydroxyphenylacetate in the absence of an inhibitor. Error bars represent one standard deviation.

The ability of phenylacetate analogs (all of which were amides) to inhibit phenylacetate decarboxylase activity was tested in cell-free extracts of the enrichment culture amended with equimolar (1 mM) concentrations of phenylacetate and the candidate inhibitor. Phenylacetamide and its *para*-hydroxy form [2-(4-hydroxyphenyl)acetamide] were equally effective inhibitors of toluene production, reducing activity to 50–55% of the activity in the absence of the inhibitor (Fig. 5). Notably, 2-(4-hydroxyphenyl)acetamide had a nearly identical effect on *p*-hydroxyphenylacetate decarboxylase and phenylacetate decarboxylase activity (Fig. 5). Phenaceturic acid (structure shown in Fig. 5) was more inhibitory than phenylacetamide (more than 70% inhibition). Atenolol, which has a structure like phenylacetamide but with a long alkyl ether substitution in *para* position on the aromatic ring, was not inhibitory at all, suggesting that the aromatic ring of phenylacetate may need to access the binding pocket of the enzyme.

## Discussion

Anaerobic biosynthesis of toluene from phenylacetate and related aromatic compounds was reported more than two decades ago, but the biochemistry underlying this novel metabolism has never been elucidated or, to our knowledge, even subjected to study. We have conducted *in vitro* characterization studies of a novel phenylacetate decarboxylase in a sewage-derived, toluene-producing enrichment culture. Among the noteworthy findings is that this enzyme is apparently not the known *p*-hydroxyphenylacetate decarboxylase (CsdBC), which we showed is unable to catalyze phenylacetate decarboxylation (Supplementary Fig. S2) and was never detected in FPLC fractions with phenylacetate decarboxylase activity (Supplementary Table S2). However, the phenylacetate decarboxylase appears to be able to catalyze both phenylacetate and *p*-hydroxyphenylacetate decarboxylation. Observations suggesting that phenylacetate and *p*-hydroxyphenylacetate decarboxylase in complex cell-free extracts are the same enzyme include the following: (i) the specific activity for both substrates is comparable in cell-free extracts (Figs 3 and 4), (ii) the activities show identical behavior during chromatographic separation of the cell-free extract on a hydroxyapatite column (Fig. 3), (iii) both activities are irreversibly inactivated upon exposure to O_2_ (Supplementary Fig. S3), and (iv) both activities are similarly inhibited by an amide analog of *p*-hydroxyphenylacetate, i.e., 2-(4-hydroxyphenyl)-acetamide (Fig. 5).

**Figure 6:**
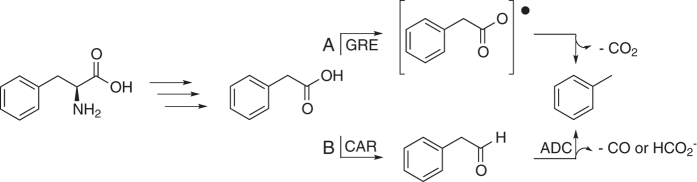
Possible routes to toluene from L-phenylalanine through the intermediate phenylacetate. Route (**A**) glycyl radical enzyme (GRE)-mediated route to initiate H atom abstraction and decarboxylation to form toluene. Route (**B**) two-step mechanism in which phenylacetate is first reduced to phenylacetaldehyde by a carboxylic acid reductase (CAR) and then decarbonylated by an aldehyde decarbonylase (ADC). As shown, formate can be a product of an aldehyde decarbonylase reaction (see text).

Our characterization of the phenylacetate decarboxylase activity allows for some speculation about the nature of the reaction. Here, we discuss what we consider to be the two most plausible toluene biosynthesis pathways. The first route is similar to that employed in *p*-cresol production catalyzed by CsdBC and HpdBC, which proceeds by way of a Kolbe-type decarboxylation (Fig. 6, Route A)^10^,^11^. The second pathway is analogous to a two-step decarboxylation described for fatty acids. This latter pathway involves the reduction of a fatty acid (as a free acid or acyl carrier protein-bound substrate) to the terminal aldehyde^16^,^17^, which is further decarbonylated in a second enzymatic step to generate the corresponding alkane and formic acid^16^,^17^,^18^ (Fig. 6, Route B). Given that both pathways entail chemically plausible mechanisms to account for toluene formation, we assess their relative likelihood in light of existing data.

Previous studies with CsdBC and HpdBC revealed the ability of these enzymes to decarboxylate *p*-hydroxyphenylacetate and form *p*-cresol^7^,^8^,^9^. Our investigations confirmed these findings and further established the inability of CsdBC from *C. scatologenes* to convert phenylacetate to toluene. While this does not rule out a radical decarboxylation mechanism, it does indicate that CsdBC and HpdBC are not properly tuned to perform the more energetically demanding decarboxylation of phenylacetate, as compared to *p*-hydroxyphenylacetate. Interestingly, we did find that an enzyme present in the complex cell-free extracts was able to decarboxylate both *p*-hydroxy-substituted and unsubstituted phenylacetate. Considering that the toluene synthase can act on phenylacetate, which lacks the resonance stabilization afforded by the *p*-hydroxy moiety of *p*-hydroxyphenylacetate (the native substrate for CsdBC and HpdBC), it is not surprising that we observed the more facile decarboxylation of *p*-hydroxyphenylacetate (Fig. 3). In support of this reaction proceeding by a mechanism analogous to that of *p*-hydroxyphenylacetate decarboxylase, we established that *p*-hydroxyphenylacetamide, a known inhibitor of HpdBC^7^, similarly inhibited the phenylacetate decarboxylase under study (Fig. 5). Moreover, the phenylacetate decarboxylase under study exhibited irreversible inactivation upon exposure to O_2_ (Supplementary Fig. S3), which is consistent with glycyl radical enzymes, including *p*-hydroxyphenylacetate decarboxylase^14^.

To investigate the possibility of the second proposed (decarbonylase) route, we focused on the second reaction, decarbonylation of phenylacetaldehyde, to determine if this conversion was catalyzed by our cell-free extracts. Incubation of the cell-free extract with phenylacetaldehyde resulted in no detectable formation of toluene (Fig. 4). Furthermore, we observed that toluene formation only proceeded under strictly anaerobic conditions; given that known aldehyde decarbonylases require oxygen^19^ and that we observed no product from phenylacetaldehyde, this mechanistic route appears to be very unlikely.

Building on the foundational studies presented here, we are actively pursuing identification of the novel phenylacetate decarboxylase in our enrichment culture, which will allow us to elucidate the mechanism by which it catalyzes the energetically challenging decarboxylation of phenylacetate. This will enable, for the first time, biochemical production of the industrially important chemical toluene from cellulosic biomass in a renewable fashion.

## Materials and Methods

### Chemicals

The chemicals used in this study were of the highest purity available and were used as received; aromatic compounds are listed in the Supplementary Material (Text S1). Highly purified water (18 MΩ resistance) obtained from a Barnstead Nanopure system (Thermo Scientific, Waltham, MA) was used to prepare all aqueous solutions described in this article.

### Cultivation of *Tolumonas auensis*, *Clostridium aerofoetidum*, *Clostridium scatologenes*, and sewage-derived, anaerobic enrichment cultures

Unless stated otherwise, all experiments were conducted under strictly anaerobic conditions in an anaerobic glove box (Type B, Coy Laboratory Products, Inc., Grass Lake, Mich.) with a nominal gas composition of 85% N_2_ – 10% CO_2_ – 5% H_2_ (ultra-high purity, anaerobic mixture).

*T. auensis* strain TA 4 (DSM 9187) was purchased from the German culture collection DSMZ (Leibniz-Institut DSMZ - Deutsche Sammlung von Mikroorganismen und Zellkulturen) and was cultivated under both anaerobic and aerobic conditions to test for toluene production using growth media and cultivation conditions described by Fischer-Romero *et al*.^5^, who isolated this strain and reported its biosynthesis of toluene. Growth media that were tested included versions of TP (toluene production) medium designed for anaerobic and aerobic cultivation, as well as DSMZ Medium 500, which has also been used successfully for anaerobic cultivation of this strain^12^. Phenylalanine and/or phenylacetate (400 μM) were used as toluene precursors in toluene production experiments.

For tests of toluene biosynthesis, *Clostridium aerofoetidum* (NCTC 505; obtained from the National Collection of Type Cultures, Salisbury, UK) was incubated for 5 days at 37 °C as described by Pons *et al*.^6^ in anaerobic thioglycollate medium (catalog #70157; Sigma Aldrich, St. Louis, MO) amended with phenylacetate. *C. aerofoetidum* strain WS described by Pons *et al*.^6^ was no longer available from culture collections at the Pasteur Institute (Paris, France) and it is not known whether the available NCTC strain is identical to strain WS.

In preparation for *in vitro* experiments testing CsdBC activity, *C. scatologenes* (ATCC 25775) was grown with ATCC medium 2107 that was prepared under strictly anaerobic conditions; 2 mM dithiothreitol (DTT) and 1 mM each of phenylacetate and *p*-hydroxyphenylacetate were added immediately before incubation for 24 hr at 37 °C.

An anaerobic, sewage-derived enrichment culture was initiated by inoculating the growth medium described in the Supplementary Material (Text S1) with sludge from the East Bay Municipal Utility District wastewater treatment plant (Oakland, CA); the culture was incubated at room temperature in an anaerobic glove box. The growth medium was modified from the TP medium described by Fischer-Romero *et al*. (1996) and was prepared using anaerobic techniques^20^; modifications included substituting bicarbonate buffer with organic buffer (HEPES, 18 mM) and reducing the sulfate concentration from 2 mM to 0.4 mM to limit sulfide production. The culture was maintained by replacing 75% of the volume with fresh medium approximately every 3 weeks. The experiments reported here were conducted after approximately 50 transfers of the culture.

### Characterization of sewage-derived, anaerobic enrichment cultures by next-generation sequencing of the metagenome and PCR-amplified 16S rRNA genes

The following methods are described in the Supplementary Material (Text S1): extraction of genomic DNA from toluene-producing enrichment cultures; construction, sequencing, and assembly of PacBio 10-kb and Illumina 270-bp and 4-kb (long mate pair) libraries; metagenome annotation; and PCR amplification, library construction, Illumina sequencing, and data analysis for 16S rRNA gene amplicons (iTags).

### Anaerobic cell suspension experiments with enrichment cultures

All steps of cell suspension experiments were conducted under anaerobic conditions. After incubating overnight with 200 μM unlabeled phenylacetate, 4.5 L of the enrichment culture was harvested anaerobically by centrifugation (13,400× *g*, 4 °C, 10 min), resuspended in 50 mL of anaerobic 10 mM sodium phosphate buffer (pH 7.5) in the anaerobic glove box, and 17 mL of the cell suspension (16.4 mg protein) was distributed into duplicate 20-mL glass vials sealed with 24-mm diameter polytetrafluoroethylene (PTFE) Mininert screw-cap valves (Sigma-Aldrich). Each vial was amended with 200 μM of labeled phenylacetate (phenylacetic acid-2-^13^C). Gaseous (static headspace) and 150 μL liquid samples were taken at time zero and every 30 min thereafter up to 210 min. Headspace samples (100 μL) were taken with a 500-μL gastight syringe (Sample-Lok Series A-2; Sigma-Aldrich) and analyzed immediately by gas chromatography/mass spectrometry (GC/MS) as described in the Supplementary Material (Text S1). Liquid samples for liquid chromatography/mass spectrometry (LC/MS) were centrifuged to remove whole cells, 100 μL of supernatant was diluted 50% with high-purity methanol, and these samples were stored in sealed glass vials with 250-μL glass inserts at 4 °C until LC/MS analysis (described in the Supplementary Material, Text S1). Analogous experiments were performed with labeled phenylalanine (L-phenylalanine-β-^13^C) substituting for labeled phenylacetate.

### Production of anaerobic cell-free extracts from enrichment cultures

Cell-free extracts were used directly in *in vitro* assays and were also subjected to FPLC separation for partial purification of phenylacetate decarboxylase activity. To make cell-free extracts, cells were harvested anaerobically as described previously and were resuspended in 10 mM sodium phosphate buffer (pH 7.5) (typically 8 mL) containing 2 mM dithiothreitol (DTT). Lysozyme (1 mg/mL final concentration) and protease inhibitors (Protease Inhibitor Cocktail Tablets, Roche) were added to the resuspended cells and the cells were incubated on ice for 10 min in an anaerobic glove box. Cells were then passed three times through a French pressure cell (138 MPa) under anaerobic conditions^21^. The lysed cells were then centrifuged (150,000× *g*, 4 °C, 15 min; Optima L-90 K Ultracentrifuge with 70.1 Ti rotor; Beckman Coulter, Indianapolis, IN) under anaerobic conditions and the supernatant was kept on ice in an anaerobic glove box until use (typically within minutes of preparation).

### Anaerobic *in vitro* assays for phenylacetate decarboxylase activity

*In vitro* assays with cell-free extracts were conducted under anaerobic conditions in an anaerobic glove box. All glass and plastic materials used to contain or manipulate assay components were allowed to degas in the glove box for at least 1 day before use. For studies of the enrichment culture, the culture was amended with 200 μM phenylactate the day before the assay. Assays were performed in 4-mL glass vials sealed with 13-mm diameter PTFE Mininert screw-cap valves (Sigma-Aldrich). The assay buffer was anaerobic 10 mM sodium phosphate buffer (pH 7.5) amended with 2 mM DTT and 1 mM phenylacetic acid-2-^13^C (except as described previously). The total liquid volume was 3.5 mL. Assay duration varied, but was typically 1 or 2 hr with gentle shaking. Headspace samples (100 μL) were taken with a 500-μL gastight syringe and analyzed by GC/MS as described in the Supplementary Material (Text S1).

### Partial purification of phenylacetate decarboxylase activity with FPLC

FPLC fractionation was performed in an anaerobic globe box. Crude cell-free extract supernatants were applied to a Bio-Scale Mini CHT-II ceramic hydroxyapatite column (5-mL bed volume, 40-μm particle diameter; Bio-Rad, Hercules, CA, USA) with a Bio-Rad Econo Gradient Pump. The chromatographic conditions were as follows: using a flow rate of 1 mL/min and a binary eluent system of 10 mM and 500 mM potassium phosphate buffer (pH 7.5; also containing 2 mM DTT), the column was held at an initial phosphate concentration of 10 mM for 1 column volume and protein was then eluted with a linear gradient from 10 to 500 mM phosphate at a rate of 49 mM/mL. One-mL fractions were collected with a model 2110 fraction collector (Bio-Rad) and assayed for phenylacetate decarboxylase activity (as described above). Attempts were made to further purify toluene synthase activity with a quaternary ammonium anion exchange column (Bio-Scale Mini UNOsphere Q) after buffer exchange of active fractions from the hydroxyapatite column, however, these attempts were unsuccessful and are not further reported here.

### Proteomic analysis of FPLC fractions by LC/MS/MS

Details on proteomic analysis of selected FPLC fractions, including data processing, are provided in the Supplementary Material (Text S1). Briefly, proteomic LC/MS/MS analysis was performed with a Q Exactive Orbitrap mass spectrometer (Thermo Scientific; Waltham, MA) in conjunction with a Proxeon Easy-nLC II HPLC (Thermo Scientific) and Proxeon nanospray source.

### Analysis of aromatic compounds by GC/MS and LC/MS

Methods used for analysis of aromatic compounds (primarily toluene, *p*-cresol, and phenylacetate) by electron-ionization GC/MS (static headspace or liquid injection) or electrospray-ionization LC/MS are detailed in the Supplementary Material (Text S1).

### Nucleotide sequence accession numbers

The metagenome sequence of the toluene-producing enrichment culture described in this study is available on JGI’s IMG-M site (https://img.jgi.doe.gov/cgi-bin/mer/main.cgi) as IMG Taxon ID 3300001784.

## Additional Information

**How to cite this article**: Zargar, K. *et al*. *In vitro* Characterization of Phenylacetate Decarboxylase, a Novel Enzyme Catalyzing Toluene Biosynthesis in an Anaerobic Microbial Community. *Sci. Rep*. **6**, 31362; doi: 10.1038/srep31362 (2016).

## Supplementary Material

Supplementary Information

## References

[b1] Mohite S. In Platts 2nd Annual Aromatics Summit 2014 (Platts McGraw Hill Financial, Seoul, Korea, 2014).

[b2] Jüttner F. & Henatsch J. J. Anoxic hypolimnion is a significant source of biogenic toluene. Nature 323, 797–798 (1986).

[b3] Jüttner F. Formation of toluene by microorganisms from anoxic freshwater sediments. Fresenius J. Anal. Chem. 339, 785–787 (1991).

[b4] Mrowiec B., Suschka J. & Keener T. C. Formation and biodegradation of toluene in the anaerobic sludge digestion process. Water Environ. Res. 77, 274–278 (2005). 15969293 10.2175/106143005x41852

[b5] Fischer-Romero C., Tindall B. J. & Juttner F. *Tolumonas auensis* gen. nov., sp. nov., a toluene-producing bacterium from anoxic sediments of a freshwater lake. Int. J. Syst. Bacteriol. 46, 183–188, doi: 10.1099/00207713-46-1-183 (1996). 8573493

[b6] Pons J. L., Rimbault A., Darbord J. C. & Leluan G. [Biosynthesis of toluene in *Clostridium aerofoetidum* strain WS]. Ann. Microbiol. (Paris) 135B, 219–222 (1984). 6508079

[b7] Selmer T. & Andrei P. I. *p*-Hydroxyphenylacetate decarboxylase from *Clostridium difficile*. A novel glycyl radical enzyme catalysing the formation of *p*-cresol. Eur. J. Biochem. 268, 1363–1372 (2001). 11231288 10.1046/j.1432-1327.2001.02001.x

[b8] Yu L., Blaser M., Andrei P. I., Pierik A. J. & Selmer T. 4-Hydroxyphenylacetate decarboxylases: properties of a novel subclass of glycyl radical enzyme systems. Biochemistry 45, 9584–9592, doi: 10.1021/bi060840b (2006). 16878993

[b9] Andrei P. I., Pierik A. J., Zauner S., Andrei-Selmer L. C. & Selmer T. Subunit composition of the glycyl radical enzyme *p*-hydroxyphenylacetate decarboxylase. A small subunit, HpdC, is essential for catalytic activity. Eur. J. Biochem. 271, 2225–2230, doi: 10.1111/j.1432-1033.2004.04152.x (2004). 15153112

[b10] Martins B. M. . Structural basis for a Kolbe-type decarboxylation catalyzed by a glycyl radical enzyme. J. Am. Chem. Soc. 133, 14666–14674, doi: 10.1021/ja203344x (2011). 21823587

[b11] Feliks M., Martins B. M. & Ullmann G. M. Catalytic mechanism of the glycyl radical enzyme 4-hydroxyphenylacetate decarboxylase from continuum electrostatic and QC/MM calculations. J. Am. Chem. Soc. 135, 14574–14585, doi: 10.1021/ja402379q (2013). 24028464

[b12] Chertkov O. . Complete genome sequence of *Tolumonas auensis* type strain (TA 4). Stand. Genomic Sci. 5, 112–120, doi: 10.4056/sigs.2184986 (2011). 22180815 PMC3236046

[b13] Carmona M. . Anaerobic catabolism of aromatic compounds: a genetic and genomic view. Microbiol. Mol. Biol. Rev. 73, 71–133, doi: 10.1128/MMBR.00021-08 (2009). 19258534 PMC2650882

[b14] Selmer T., Pierik A. J. & Heider J. New glycyl radical enzymes catalysing key metabolic steps in anaerobic bacteria. Biol. Chem. 386, 981–988, doi: 10.1515/BC.2005.114 (2005). 16218870

[b15] Heider J., Spormann A. M., Beller H. R. & Widdel F. Anaerobic bacterial metabolism of hydrocarbons. FEMS Microbiology Reviews 22, 459–473 (1998).

[b16] Schirmer A., Rude M. A., Li X., Popova E. & del Cardayre S. B. Microbial biosynthesis of alkanes. Science 329, 559–562, doi: 10.1126/science.1187936 (2010). 20671186

[b17] Akhtar M. K., Turner N. J. & Jones P. R. Carboxylic acid reductase is a versatile enzyme for the conversion of fatty acids into fuels and chemical commodities. Proc. Natl. Acad. Sci. USA 110, 87–92, doi: 10.1073/pnas.1216516110 (2013). 23248280 PMC3538209

[b18] Li N. . Conversion of fatty aldehydes to alka(e)nes and formate by a cyanobacterial aldehyde decarbonylase: cryptic redox by an unusual dimetal oxygenase. J. Am. Chem. Soc. 133, 6158–6161, doi: 10.1021/ja2013517 (2011). 21462983 PMC3113487

[b19] Li N. . Evidence for only oxygenative cleavage of aldehydes to alk(a/e)nes and formate by cyanobacterial aldehyde decarbonylases. Biochemistry 51, 7908–7916, doi: 10.1021/bi300912n (2012). 22947199

[b20] Beller H. R., Legler T. C. & Kane S. R. Genetic manipulation of the obligate chemolithoautotrophic bacterium *Thiobacillus denitrificans*. Methods Mol. Biol. 881, 99–136, doi: 10.1007/978-1-61779-827-6_5 (2012). 22639212

[b21] Beller H. R. & Spormann A. M. Substrate range of benzylsuccinate synthase from *Azoarcus* sp. strain T. FEMS Microbiol. Lett. 178, 147–153 (1999). 10483734 10.1111/j.1574-6968.1999.tb13771.x

