# Supplementary material for: In vitro Characterization of Phenylacetate Decarboxylase, a Novel Enzyme Catalyzing Toluene Biosynthesis in an Anaerobic Microbial Community
Source: Sci Rep. 2016 Aug 10;6:31362. doi: 10.1038/srep31362 (PMC4979209; doi:10.1038/srep31362)
Supplement: Supplementary Information [file srep31362-s1.pdf]

SUPPLEMENTARY INFORMATION

In vitro Characterization of Phenylacetate Decarboxylase, a Novel Enzyme Catalyzing Toluene Biosynthesis in an Anaerobic Microbial Community

K. Zargar[1], R. Saville[1], R. Phelan[1,2], S. G. Tringe[3], C. J. Petzold[1,4], J. D. Keasling[1, 4,5,6], H. R. Beller* [1,7]

Joint BioEnergy Institute (JBEI), 5885 Hollis Avenue, Emeryville, CA, USA[1]; QB3 Institute, University of California, Berkeley, California 94270, United States[2]; Joint Genome Institute, 2800 Mitchell Drive, Walnut Creek, CA, USA[3]; Biological Systems and Engineering, Lawrence Berkeley National Laboratory (LBNL), Berkeley, CA, USA[4]; Departments of Chemical & Biomolecular Engineering and of Bioengineering, University of California, Berkeley, CA, USA[5]; Novo Nordisk Foundation Center for Biosustainability, Technical University of Denmark, Kogle Allé, DK2970-Hørsholm, Denmark[6]; Earth and Environmental Sciences, LBNL, Berkeley, CA, USA[7]

SUPPLEMENTARY INFORMATION

TEXT S1. AUXILIARY MATERIALS AND METHODS

Aromatic chemicals

Aromatic compounds included the following: toluene (Sigma-Aldrich, St. Louis, MO; $\geq$99.9%), phenylacetic acid (Aldrich; 99%), phenylacetic acid-2-^{13}C (Icon Isotopes, Summit, NJ; 99 atom% ^{13}C), L-phenylalanine-β-^{13}C (Aldrich; 99 atom% ^{13}C), *p*-hydroxyphenylacetic acid (Aldrich, 98%), *p*-cresol (Fluka, St. Louis, MO; $\geq$99.7%), 2-phenylpropionate (Aldrich; 97%), 3-phenylpropionate (Sigma-Aldrich; 99%), phenylacetaldehyde (Alfa Aesar, Ward Hill, MA; 95%), 2-phenylacetamide (Oakwood Chemical, Estill, SC; 99%), 2-(4-hydroxyphenyl)acetamide (Aldrich; 99%), phenaceturic acid (TCI America, Portland, OR; >98%), atenolol (Sigma; $\geq$98%), ethylbenzene (Fluka; $\geq$99.5%), and 4-ethyltoluene (Fluka; $\geq$95%).

Growth medium for sewage-derived enrichment culture

The growth medium (pH 7.1) for the enrichment culture included the following compounds (grams per liter): KH_2PO_4 (0.25), NH_4Cl (0.34), KCl (0.34), sodium HEPES (4.69), yeast extract (0.01), glucose (1), $MgCl_2 \cdot 6H_2O$ (1), $MgSO_4 \cdot 7H_2O$ (0.1), $CaCl_2 \cdot 2H_2O$ (0.125), and vitamin B_{12} (2×10^{-5}); 0.5 mL of trace element solution (1) was added per liter of medium. The trace element solution contained the following compounds in 100 mL of solution: 7.7 N (25%) HCl (1.25 mL), $FeSO_4 \cdot 7H_2O$ (210 mg), $MnCl_2 \cdot 4H_2O$ (10 mg), $CoCl_2 \cdot 6H_2O$ (19 mg), $ZnCl_2$ (7 mg), $NiCl_2$ (1.3 mg), $CuCl_2 \cdot 2H_2O$ (0.2 mg), $Na_2MoO_4 \cdot 2H_2O$ (3.6 mg), and H_3BO_3 (0.6 mg).

Characterization of sewage-derived, anaerobic enrichment cultures by next-generation sequencing of the metagenome and PCR-amplified 16S rRNA genes

Extraction of genomic DNA from toluene-producing enrichment cultures was performed with a bead-beating method involving hexadecyltrimethylammonium bromide (CTAB) extraction buffer described elsewhere (2). Genomic DNA was purified with Allprep DNA/RNA kits (Qiagen, Valencia, CA).

Metagenome analysis

Construction, sequencing, and assembly of Illumina 270-bp and 4-kb (long mate pair) libraries and PacBio 10-kb libraries are described below:

<u>NHPP – Illumina Regular Fragment, 270bp:</u>
100 ng of DNA was sheared to 270 bp using the Covaris LE220 (Covaris) and size selected using SPRI beads (Beckman Coulter). The fragments were treated with end-repair, A-tailing, and ligation of Illumina compatible adapters (IDT, Inc) using the KAPA-Illumina library creation kit (KAPA Biosystems). The prepared library was then quantified using KAPA Biosystems' next-generation sequencing library qPCR kit and run on a Roche LightCycler 480 real-time PCR instrument. The quantified library was then prepared for sequencing on the Illumina HiSeq sequencing platform utilizing a TruSeq paired-end cluster kit, v3, and Illumina's

cBot instrument to generate a clustered flowcell for sequencing. Sequencing of the flowcell was performed on the Illumina HiSeq2000 sequencer using a TruSeq SBS sequencing kit 200 cycles, v3, following a 2x150 indexed run recipe.

<u>NHUT – Illumina Regular LMP, 4kb, CLRS:</u>
5-10 µg of DNA was sheared using the Covaris g-TUBE™ (Covaris) and gel size selected for 4 kb. The sheared DNA was treated with end repair and ligated with biotinylated adapters containing *loxP*. The adapter ligated DNA fragments were circularized via recombination by a Cre excision reaction (NEB). The circularized DNA templates were then randomly sheared using the Covaris LE220 (Covaris). The sheared fragments were treated with end repair and A-tailing using the KAPA-Illumina library creation kit (KAPA Biosystems) followed by immobilization of mate pair fragments on streptavidin beads (Invitrogen). Illumina compatible adapters (IDT, Inc) were ligated to the mate pair fragments and 8 cycles of PCR were used to enrich for the final library (KAPA Biosystems). The prepared library was then quantified using KAPA Biosystems' next-generation sequencing library qPCR kit and run on a Roche LightCycler 480 real-time PCR instrument. The quantified library was then prepared for sequencing on the Illumina HiSeq sequencing platform utilizing a TruSeq paired-end cluster kit, v3, and Illumina's cBot instrument to generate a clustered flowcell for sequencing. Sequencing of the flowcell was performed on the Illumina HiSeq2000 sequencer using a TruSeq SBS sequencing kit 200 cycles, v3, following a 2x150 indexed run recipe.

<u>PB0742 – LC - PacBio 10kb:</u>
Unamplified libraries were generated using Pacific Biosciences standard template preparation protocol for creating 10-kb libraries. 5 µg of gDNA was used to generate the library and the DNA was sheared using a Covaris g-TUBE™ to generate sheared fragments of 10 kb in length. The sheared DNA fragments were then prepared using Pacific Biosciences SMRTbell template preparation kit, where the fragments were treated with DNA damage repair, had their ends repaired so that they were blunt-ended, and 5' phosphorylated. Pacific Biosciences hairpin adapters were then ligated to the fragments to create the SMRTbell template for sequencing. The SMRTbell templates were then purified using exonuclease treatments and size-selected using AMPure PB beads. Sequencing primer was then annealed to the SMRTbell templates and Version P4 sequencing polymerase was bound to them. The prepared SMRTbell template libraries were then sequenced on a Pacific Biosciences RSII sequencer using Version C2 chemistry and 2-hr sequencing movie run times.

Metagenome assembly was carried out as described in Appendix A of this document. Annotation and genome analysis was performed through JGI's IMG/M 4 system (3).

Microbial community analysis (16S rRNA gene iTags)

Analysis of microbial community composition based upon Illumina sequencing of 16S rRNA gene amplicons (iTags) is described below and data analysis is documented in Appendix B of this document.

Community DNA samples were received in a 96-well plate for generation of 16S V4 rRNA amplicon libraries for Illumina sequencing. Sample preparation was performed on a PerkinElmer Sciclone NGS G3 Liquid Handling Workstation capable of processing 96 plate-based samples in parallel, utilizing 5 PRIME's HotMasterMix amplification kit and custom amplification primers targeting the V4 region of the 16S rRNA gene. Primers also contained the Illumina sequencing adapter sequence and a unique barcode index sequence specific to each well on the plate, which allowed for multiplexing of prepared amplicons and direct sequencing. Prepared amplicon libraries were then normalized and multiplexed into a single pool of amplicons for the entire plate. The prepared pool of 16S V4 rRNA amplicon libraries were then quantified using KAPA Biosystems' next-generation sequencing library qPCR kit and run on a Roche LightCycler 480 real-time PCR instrument. The quantified pool was then loaded on an Illumina MiSeq sequencer using v3 reagent kit and a 2x300 indexed run recipe.

Dialysis for anaerobic *in vitro* assays for phenylacetate decarboxylase activity

A variety of studies were conducted using *in vitro* assays, including dialysis experiments (Figure HB2). These were conducted with 10-mL D-tube dialyzers (molecular weight cutoff = 3.5 kDa; Novagen, EMD Millipore, Billerica, MA) and a 2-L reservoir of 10 mM sodium phosphate (pH 7.5) as the dialysis buffer. Dialysis was allowed to proceed for 8 hr on ice with constant stirring. All dialyzed controls and samples used 1 mL of dialyzed lysate from the same dialysis tube; undialyzed lysates for controls were kept on ice for 8 hr to be comparable with the dialyzed samples. Dialysis was shown to be effective by independent trials that contained lysate amended with sodium bromide tracer; these trials demonstrated that 99% of the bromide was removed within 4 hr of dialysis.

Shotgun proteomic analysis of FPLC fractions by LC-MS/MS

Extraction and tryptic digestion

Proteins in selected FPLC fractions were processed for proteomic analysis as previously described (4). Briefly, the proteins were extracted by chloroform/methanol precipitation and resuspended in 100 mM ammonium bicarbonate with 20% acetonitrile. The proteins were reduced with tris(2-carboxyethyl)phosphine (TCEP) for 30 min, followed by incubation with iodoacetamide (IAA; final conc. 10 mM) for 30 min in the dark, and then digested overnight with MS-grade trypsin (1:50 w/w trypsin: protein) at 37°C.

LC-MS/MS analysis

Digested peptides were analyzed by LC-MS/MS on a Thermo Scientific Q Exactive Orbitrap Mass spectrometer in conjunction with a Proxeon Easy-nLC II HPLC (Thermo Scientific) and Proxeon nanospray source. The digested peptides were loaded onto a 100-µm x 25-mm Magic C18 100Å 5U reverse phase trap column where they were desalted online before being separated using a 75-µm x 150-mm Magic C18 200Å 3U reverse phase column. Peptides were eluted using a 90-min gradient with a flow rate of 300 nL/min. An MS survey scan was obtained for the *m/z* range 300-1600, MS/MS spectra were acquired using a "top 15" method, where the top 15 ions in the MS spectra were subjected to HCD (High Energy Collisional

Dissociation). An isolation mass window of 2.0 *m/z* was for the precursor ion selection, and normalized collision energy of 27% was used for fragmentation. A 5-sec duration was used for the dynamic exclusion.

Tandem mass spectra were extracted and charge state deconvoluted by Proteome Discoverer (Thermo Scientific). All MS/MS samples were analyzed using X! Tandem (The GPM, thegpm.org; version TORNADO (2013.02.01.1)). X! Tandem was set up to search a database comprising FASTA translated sequences from the toluene-producing metagenome (IMG Taxon ID 3300001784), the cRAP database of common laboratory contaminants (www.thegpm.org/crap; 114 entries), plus an equal number of reverse protein sequences assuming the digestion enzyme trypsin. X! Tandem was searched with a fragment ion mass tolerance of 20 ppm and a parent ion tolerance of 20 ppm. Iodoacetamide derivative of cysteine was specified in X! Tandem as a fixed modification. Deamidation of asparagine and glutamine, oxidation of methionine and tryptophan, methionine oxidation to sulfone, tryptophan oxidation to formylkynurenine of tryptophan, and acetylation of the N-terminus were specified in X! Tandem as variable modifications.

Criteria for protein identification

Scaffold (version Scaffold_4.4.1, Proteome Software Inc., Portland, OR) was used to validate MS/MS-based peptide and protein identifications. Peptide identifications were accepted if they could be established at greater than 85.0% probability by the Scaffold Local FDR algorithm. Protein identifications were accepted if they could be established at greater than 80.0% probability to achieve an FDR less than 5.0% and contained at least 1 identified peptide. Protein probabilities were assigned by the Protein Prophet algorithm (Nesvizhskii et al., 2003, *Anal. Chem.* **75**:4646-58). Proteins that contained similar peptides and could not be differentiated based on MS/MS analysis alone were grouped to satisfy the principles of parsimony. Proteins sharing significant peptide evidence were grouped into clusters.

Analysis of aromatic compounds by GC-MS and liquid chromatography-mass spectrometry (LC-MS)

Toluene and other volatile aromatic substrates were analyzed by static headspace-electron ionization (EI) GC-MS using a model 7890A GC (Agilent, Santa Clara, CA) with a DB-5 fused silica capillary column (30-m length, 0.25-mm inner diameter, 0.25-µm film thickness; Agilent) coupled to an HP 5975C series mass selective detector. The GC oven was held isothermal at 60ºC; the injection port temperature was 250ºC, and the transfer line temperature was 280ºC. The carrier gas, ultra high-purity helium, flowed at a constant rate of 1 ml/min. Injections were manual and splitless, with the split turned on after 1 min. For labeled and unlabeled toluene, selected ion monitoring (SIM) was used to acquire data for *m/z* 91, 92, and 93 (75 msec dwell time for each ion); other volatile compounds were determined in full-scan mode (50 – 300 amu at 5.5 scans/sec). *p*-Cresol was measured similarly, except that 1-µl liquid injections of concentrated hexane extracts were performed with a model 7683B autosampler (Agilent), the MS acquired data in full-scan mode (*m/z* 50-600 amu, 2.66 scans/sec), and the temperature program was 40ºC (hold 3 min) and increase to 295ºC at 15ºC /min.

For toluene, external standard quantification was performed with 4 or 5 calibration standards that had identical vials and liquid/headspace ratios as the samples. Quantification of [*methyl*-^{13}C]toluene, which was the product in all *in vitro* and *in vivo* studies in which phenylacetate or phenylalanine was a substrate, merits additional detail. Since cells and cell-free lysates had some residual unlabeled toluene from cultivation, this had to be corrected for. Unlabeled toluene has 3 distinctive fragment ions at *m/z* 91 (100% relative abundance), *m/z* 92 (59.5%), and *m/z* 93 (4.2%). [*Methyl*-^{13}C]toluene has a spectrum that is shifted up 1 amu, so *m/z* 92 is at 100% and *m/z* 93 is at 59.5%. Since *m/z* 91 occurs only in unlabeled toluene, contributions of unlabeled toluene to the labeled toluene mass spectrum were corrected for by subtracting 59.5% of the *m/z* 91 area from *m/z* 92, and 4.2% of the *m/z* 91 area from *m/z* 93. If [*methyl*-^{13}C]toluene was present in a sample, then the ratio of the corrected *m/z* 93 area to the corrected *m/z* 92 area should have been 59.5%. Thus, the corrected *m/z* 93 area was used for [*methyl*-^{13}C]toluene quantification only if that ratio was 0.595 (within experimental error).

Analyses of ^{13}C-labeled phenylacetic acid were made with a LC/MSD SL (Agilent) equipped with a model 1260 Infinity Binary Pump and operated in the electrospray ionization, negative ion mode. The mobile phase flowed at 240 µL/min (0–5 min) or 350 µL/min (5-10 min) through a Kinetex XB-C18 column (2.6-µm particle size, 100Å, 3-mm inner diameter x 100-mm length; Phenomenex). The initial mobile phase composition was 60 vol% A (10 mM formic acid in reagent water) and 40 vol% B (10 mM formic acid in high-purity methanol), then was increased linearly to 80% B at 4 min, decreased linearly to 40% B from 4.7 to 5 min, and remained at 40% B for 5 min to allow the column to re-equilibrate to initial conditions. The sample injection volume was 10 µL. Source conditions included 3.5 kV capillary voltage, 250°C drying gas temperature, 12 L/min drying gas flow, and 241 kPa nebulizer pressure. LC/MS/MS data acquisition for ^{13}C-labeled phenylacetic acid was in the SIM mode at *m/z* 136.2. Four-point calibrations were performed for external standard quantification.

1. **Widdel F, Bak F.** 1992. Gram-negative mesophilic sulfate-reducing bacteria, p. 3352-3378. *In* Balows A, Truper HG, Dworkin M, Harder W, Schleifer K-H (ed.), The Prokaryotes. Springer-Verlag, New York.

2. **DeAngelis KM, Brodie EL, DeSantis TZ, Andersen GL, Lindow SE, Firestone MK.** 2009. Selective progressive response of soil microbial community to wild oat roots. ISME J. **3:**168-178.

3. **Markowitz VM, Chen IM, Chu K, Szeto E, Palaniappan K, Pillay M, Ratner A, Huang J, Pagani I, Tringe S, Huntemann M, Billis K, Varghese N, Tennessen K, Mavromatis K, Pati A, Ivanova NN, Kyrpides NC.** 2014. IMG/M 4 version of the integrated metagenome comparative analysis system. Nucleic Acids Res. **42:**D568-573.

4. **González Fernández-Niño SM, Smith-Moritz AM, Chan LJG, Adams PD, Heazlewood JL, Petzold CJ.** 2015. Standard flow liquid chromatography for shotgun proteomics in bioenergy research. Frontiers in Bioengineering and Biotechnology **3**.

APPENDIX A

Assembly of metagenomic libraries

JGI assembly of 1011269 NHUT_NHPP+pacbio 1011269 is complete.

Allpaths assemblies were run using different estimated genome sizes to sample different coverages using the std and LMP libraries. PBJelly was used to add PacBio data to each individual allpaths assembly. Minimus2 was used to merge all the allpaths contigs with the default metagenome pipeline soap assembly, following by sspace to scaffold contigs over 5 kb.

The assembled contigs stats are as follows:

	A	C	G	T	N	GC	GC_stdev
Base Content	0.2667	0.2331	0.2339	0.2663	0.0052	0.4670	0.0953

Main genome scaffold total: 78801
Main genome contig total: 80315
Main genome scaffold sequence total: 292.867 MB
Main genome contig sequence total: 291.373 MB 0.510% gap
Main genome scaffold N/L50: 815/54.927 KB
Main genome contig N/L50: 1184/38.846 KB
Max scaffold length: 2.197 MB
Max contig length: 1.678 MB
Number of scaffolds > 50 KB: 923
% main genome in scaffolds > 50 KB: 51.94%

Minimum Scaffold Length	Number of Scaffolds	Number of Contigs	Total Scaffold Length	Total Contig Length	Scaffold Contig Coverage
All	78,801	80,315	292,867,165	291,372,904	99.49%
50	78,801	80,315	292,867,165	291,372,904	99.49%
100	78,801	80,315	292,867,165	291,372,904	99.49%
250	74,621	76,135	291,852,385	290,358,134	99.49%
500	35,969	37,483	279,551,910	278,058,994	99.47%
1 KB	30,187	31,701	275,626,303	274,134,559	99.46%
2.5 KB	12,647	14,161	248,313,962	246,823,259	99.40%
5 KB	5,617	7,131	224,103,894	222,613,923	99.34%
10 KB	3,529	5,043	209,617,549	208,137,808	99.29%
25 KB	1,758	2,964	181,596,860	180,448,005	99.37%
50 KB	923	1,734	152,126,584	151,363,251	99.50%
100 KB	424	887	117,802,467	117,385,505	99.65%
250 KB	146	347	75,814,738	75,652,822	99.79%
500 KB	47	145	41,505,252	41,424,473	99.81%
1 MB	12	58	18,053,645	18,014,403	99.78%

Read stats are as follows:

589653354 + 9860 in total (QC-passed reads + QC-failed reads)
0 + 0 duplicates
569885297 + 0 mapped (96.65%:0.00%)
589653354 + 9860 paired in sequencing
294824248 + 7359 read1
294829106 + 2501 read2
535557564 + 0 properly paired (90.83%:0.00%)
563723540 + 0 with itself and mate mapped
6161757 + 0 singletons (1.04%:0.00%)
26850668 + 0 with mate mapped to a different chr
26850668 + 0 with mate mapped to a different chr (mapQ>=5)

If you have any questions, please let us know: Brian Foster bfoster@lbl.gov, Alex Copeland accopeland@lbl.gov

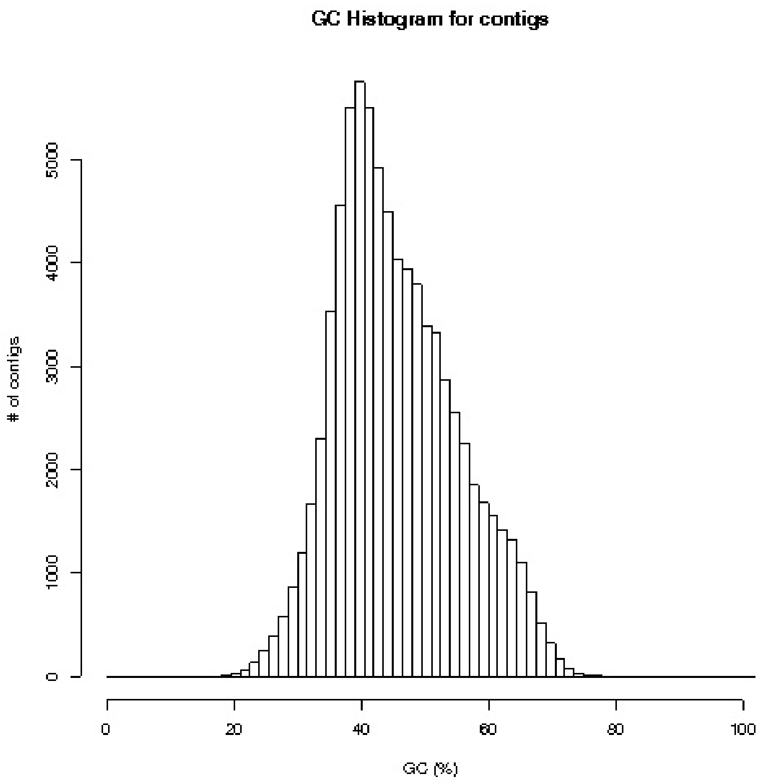
GC Histogram for contigs
# of contigs
0
1000
2000
3000
4000
5000
0
20
40
60
80
100
GC (%)

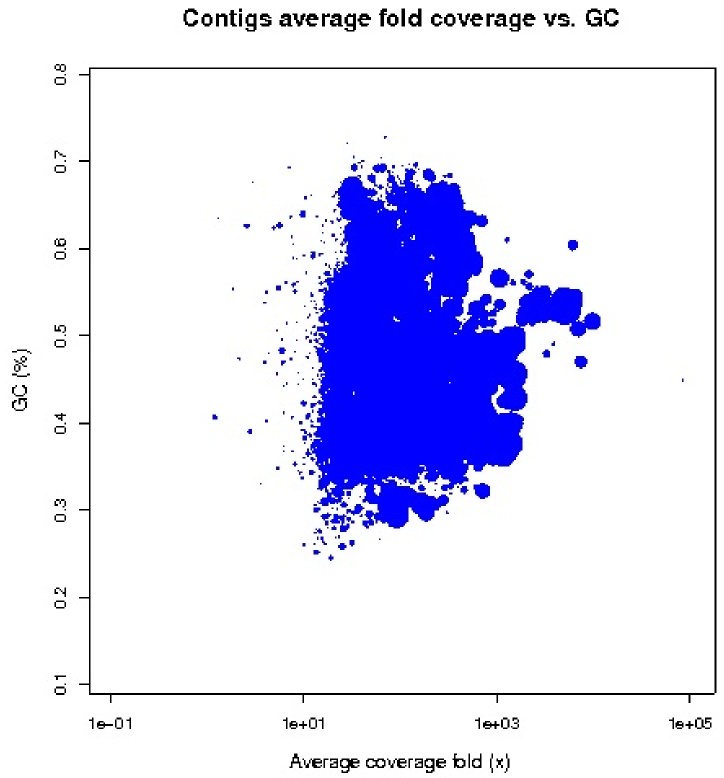
Contigs average fold coverage vs. GC
GC (%)
0.1
0.2
0.3
0.4
0.5
0.6
0.7
0.8
1e−01
1e+01
1e+03
1e+05
Average coverage fold (x)

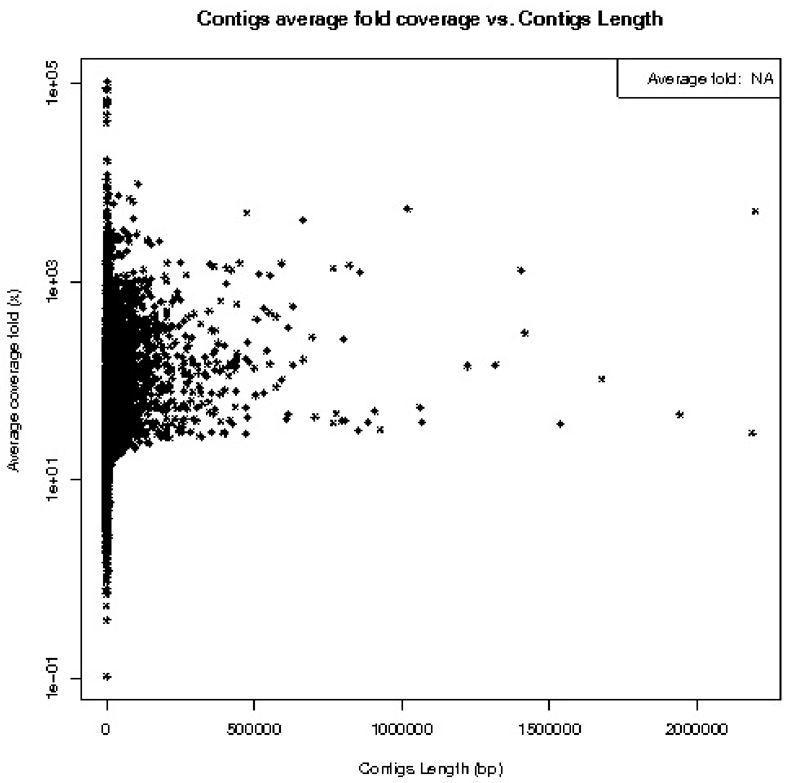

Contigs average fold coverage vs. Contigs Length
Average fold: NA
Average coverage fold (x)
1e+05
1e+03
1e+01
1e-01
0
500000
1000000
1500000
2000000
Contigs Length (bp)

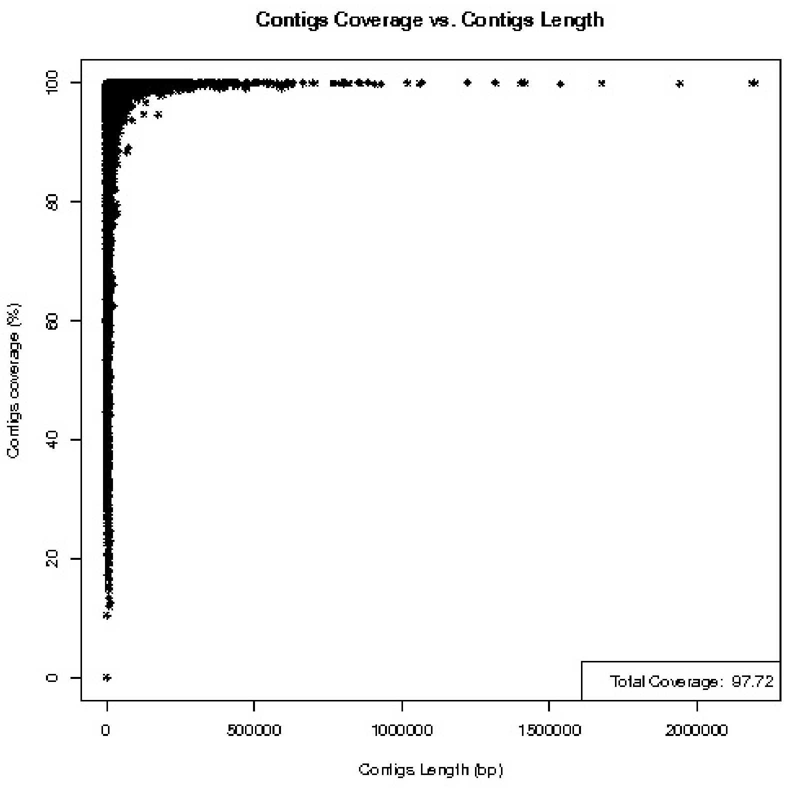

Contigs Coverage vs. Contigs Length
Contigs coverage (%)
100
80
60
40
20
0
Total Coverage: 97.72
500000
1000000
1500000
2000000
Contigs Length (bp)

APPENDIX B

Informatics of iTag analyses

iTagger v1.1 METHODS

The iTagger amplicon analysis pipeline uses several publicly available tools to analyze amplicon libraries, such as 16S rRNA or fungal ITS variable regions for phylogenetic analysis. All libraries to be compared should be identically constructed, sequenced, and analyzed.

I. INPUT:

(1) Configuration file in INI format with parameters and paths of reference databases.
   OUTPUT: The config file is copied to <OUTDIR>/config.ini
(2) Libraries tabular file indicates library/condition name and path to Fastq file.

II. READ QC:

Each library's Fastq file is processed as described below and the results are saved in the data/ folder, which also includes a readQC.log file which indicates the read-pairs at each step and the percentage of pairs which pass each step (i.e. percentages are per-step, not of total input).
   OUTPUT: Saved in <OUTDIR>/data/<LIB_NAME>/
      Summarized in readQC.log

(1) CONTAM FILTER: Filter one or more contaminants using Duk (e.g. PhiX control, sequencing library adapter dimers, human contaminants, etc.). For paired reads, the entire pair is filtered if one end-read has a high-scoring hit.
   OUTPUT: duk.log
(2) PRIMER TRIM: PCR primers (of the conserved region) are trimmed away. For paired-end reads, both forward and reverse primers must be found, otherwise the entire pair is filtered.
(3) HARD TRIM: For particular libraries (e.g. fungal ITS), it is useful to trim a predefined number of bases from the 5' and 3' ends of the sequence. For fungal ITS, we observed better RDP Classifier results after trimming conserved regions. We do not recommend hard trimming for 16S sequences.
(4) ITERATIVE PAIR MERGING: Reads are trimmed as a pair, removing the last base from whichever end has the highest expected error in a window 5bp wide. Reads are trimmed from mean + 3 standard deviations to mean - 3 standard deviations in 0.5 standard deviation steps. After each trimming step, pairs are merged into single sequences with either Flash or Pandaseq. Pairs which are not merged continue to the next round of trimming. Paired reads which are not combined are discarded.
   OUTPUT: Not-combined reads, nc.fastq.bz2
(5) EXPECTED-ERROR FILTER: Merged reads are filtered if they have an expected number of errors which exceeds the threshold. The config file indicates the maximum number of expected errors per 100bp. Note that Flash and Pandaseq produce different quality scores in the overlap-assembled regions.

OUTPUT: Filtered extended reads, ex.fastq.bz2
Quality report, qualStat.pdf, qualStat.tsv
(7) DEREPLICATE: Count the number of times each sequence is observed and output in tabular (seq-obs) format, ordered by sequence.
OUTPUT: seqobs.tsv.bz2

III. CLUSTERING:

USEARCH is used for clustering, although there is a provision for iterative clustering which can (a) provide faster processing and (b) allow processing of larger files than can normally be processed (particularly with the 32bit version). RDP Classifier is used for taxonomic classification of the resultant cluster centroid sequences and it's accuracy is dependent upon providing a well-curated RDP reference database.
OUTPUT: Saved in <OUTDIR>/otu
Summarized in cluster.log

(1) MERGE LIBRARIES: The seq-obs files for all libraries are merged, dereplicated, and sorted by decreasing abundance. Low-abundance sequences are separated and excluded from clustering, step 2 (although they will be mapped and counted in step 3).
(2) ITERATIVE CLUSTER OTUS: Refer to the USEARCH documentation for the algorithm description. Our use of USEARCH differs slightly from that described in the USEARCH documentation in that we iterate between single-threaded clustering and multi-threaded searching in order to reduce run-time. We also use .obs files for tracking cluster members, so a final mapping and counting step (as described in the USEARCH docs) is unnecessary. Clustering is done iteratively starting at 99% identity, and decreasing by 1% identity until the level described in the config file is reached (e.g. 97% for 16S, 95% for Fungal ITS).
(3) MAP LOW-ABUNDANCE SEQUENCES: Rare sequences, which cannot form their own clusters, are mapped to the cluster centroid sequences and counted.
(4) REFERENCE DB CHIMERA FILTER: Centroid sequences are compared to the reference database and likely chimeric sequences discarded, using UCHIME.
OUTPUT: Final cluster centroids, otu.fasta.bz2
(5) CLASSIFICATION: Assign taxonomic classification to each cluster using RDP Classifier. The config file indicates a taxonomic level (e.g. family) and confidence level (e.g. 0.5) which is used to decide which classifications are useful. Clusters which can be acceptably classified are output to otu.tax.tsv, while the others are written to otu.unk.tsv.
OUTPUT: RDP output, rdp.tsv
Classified OTUs, otu.tax.tsv
Unclassified OTUs, otu.unk.tsv
(6) TAX FILTER: Clusters with classifications which do not match those indicated in the config file are discarded and the desired clusters are written to the otu.tax.filtered.tsv file.
OUTPUT: Final OTU table, otu.tax.filtered.tsv

IV. TAXONOMIC ANALYSIS:

QIIME is used to manipulate the final OTUs file. A few graphs are generated plus some rarefied tables which may be useful for subsequent analysis.

(1) GENERATE BIOM: The BIOM JSON file is generated from the OTU tabular file.
OUTPUT: otu.biom
(2) ABUNDANCE THRESHOLD: Filter OTUs are assorted levels and calculate alpha diversities.
OUTPUT: Several files in <OUTDIR>/abundance_thresholds/
(3) SINGLE RAREFACTION: This is done at 1000 and at a level calculated from the trimmed mean and standard deviation of the library sizes (10% trimmed, and calc. mean - i * stdev; while i is the highest number from 2 to 0.5, step 0.5, until the cutoff is above 0).
OUTPUT: <OUTDIR>/otu/rarefied.1000.biom, rarefied.1000.filtered.biom
<OUTDIR>/otu/rarefied.<X>.biom, rarefied.<X>.filtered.biom
(4) SUMMARIZE TAXONOMY: with both relative and absolute abundance
OUTPUT: <OUTDIR>/tax_mapping/relative/
<OUTDIR>/tax_mapping/absolute/
(5) PLOT RANK-ABUNDANCE: Generate PDF rank-abundance graph of all samples.
OUTPUT: <OUTDIR>/otu/log_rank_abundance.pdf
(6) PLOT TAXA SUMMARY: Make taxa plot of absolute abundance
OUTPUT: <OUTDIR>/tax_mapping/plots/
(7) PHYLUM BARPLOT: generate a phylum-level barplot using absolute abundance for a quick overview of the data.
OUTPUT: <OUTDIR>/tax_mapping/taxonomy_phylum_L2.tab

* * *

iTagger was written by Julien Tremblay (julien.tremblay@mail.mcgill.ca) and Edward Kirton (ESKirton@LBL.gov) and is Copyright (c) 2013 by the US DOE Joint Genome Institute but is freely available for use without any warranty under the same license as Perl itself. v1.1 was released 12/12/2013. Refer to wrapped tools for their author and license information.

* * *

External executable versions:

duk: Version 1.05
cutadapt: 1.2.1
FLASH v1.2.6
pandaseq 2.5 <andre@masella.name>
usearch v7.0.959_i86linux32
RDP Classifier: /usr/common/jgi/statistics/rdp-classifier/2.5/rdp_classifier-2.5.jar
QIIME: /usr/common/jgi/frameworks/qiime/1.7.0/bin/alpha_diversity.py

SUPPLEMENTARY INFORMATION

Figures S1 – S3

In vitro Characterization of Phenylacetate Decarboxylase, a Novel Enzyme Catalyzing Toluene Biosynthesis in an Anaerobic Microbial Community

K. Zargar[1], R. Saville[1], R. Phelan[1,2], S. G. Tringe[3], C. J. Petzold[1,4], J. D. Keasling[1, 4,5,6], H. R. Beller* [1,7]

Joint BioEnergy Institute (JBEI), 5885 Hollis Avenue, Emeryville, CA, USA[1]; QB3 Institute, University of California, Berkeley, California 94270, United States[2]; Joint Genome Institute, 2800 Mitchell Drive, Walnut Creek, CA, USA[3]; Biological Systems and Engineering, Lawrence Berkeley National Laboratory (LBNL), Berkeley, CA, USA[4]; Departments of Chemical & Biomolecular Engineering and of Bioengineering, University of California, Berkeley, CA, USA[5]; Novo Nordisk Foundation Center for Biosustainability, Technical University of Denmark, Kogle Allé, DK2970-Hørsholm, Denmark[6]; Earth and Environmental Sciences, LBNL, Berkeley, CA, USA[7]

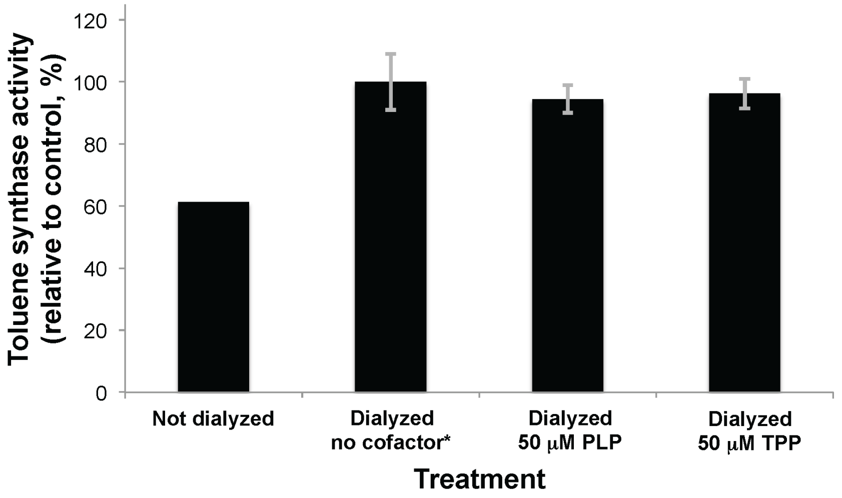

Figure S1. Phenylacetate decarboxylase activity in clarified lysates of sewage-derived enrichment cultures: undialyzed lysate, dialyzed lysate with no amendments (negative control), dialyzed lysate amended with pyridoxal-5'-phosphate (PLP), dialyzed lysate amended with thiamine pyrophosphate (TPP). PLP and TPP are common decarboxylation co-factors[1,2]. Bars are normalized to the negative control. Error bars represent one standard deviation.

1 Li, T., Huo, L., Pulley, C. & Liu, A. Decarboxylation mechanisms in biological system. *Bioorg. Chem.* **43**, 2-14, doi:10.1016/j.bioorg.2012.03.001 (2012).

2 Jordan, F. & Patel, H. Catalysis in Enzymatic Decarboxylations: Comparison of Selected Cofactor-dependent and Cofactor-independent Examples. *ACS Catal.* **3**, 1601-1617, doi:10.1021/cs400272x (2013).

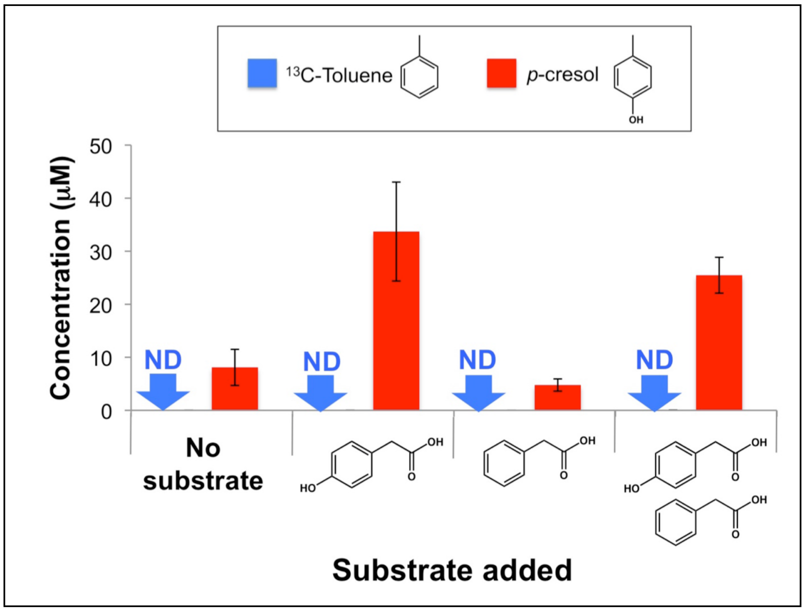

Figure S2. Toluene and *p*-cresol production from phenylacetate and *p*-hydroxyphenylacetate, respectively, in clarified cell lysates of *Clostridium scatologenes*, which natively expresses a *p*-hydroxyphenylacetate decarboxylase (CsdBC). ND, not detected. Error bars represent one standard deviation. The background level of *p*-cresol apparent in the assays is a consequence of carryover from the original *C. scatologenes* cultures, which were grown in the presence of both *p*-hydroxyphenylacetate and phenylacetate.

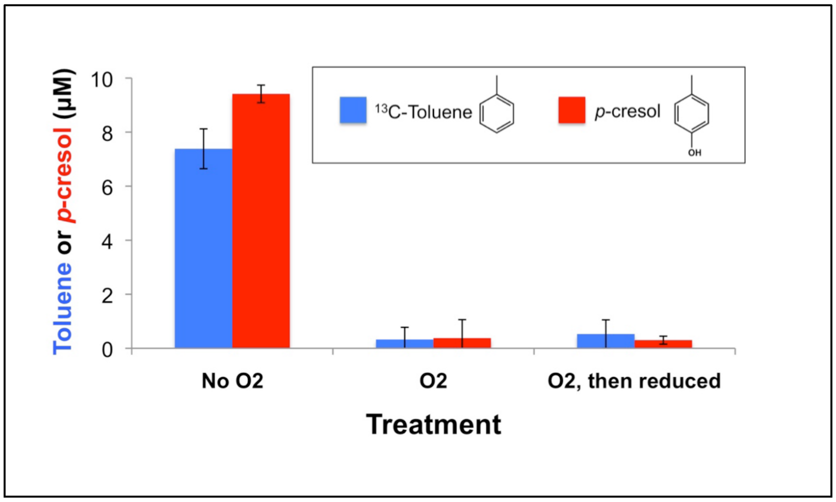

Figure S3. Toluene and *p*-cresol production from phenylacetate and *p*-hydroxyphenylacetate, respectively, in clarified lysates with no O_2 exposure, after O_2 exposure, and after O_2 exposure and subsequent reduction with dithiothreitol.

Zargar, Saville, Phelan, Tringe, Petzold, Keasling, and Beller. *In vitro* Characterization of Phenylacetate Decarboxylase, a Novel Enzyme Catalyzing Toluene Biosynthesis in an Anaerobic Microbial Community, *Scientific Reports*

Table S1. Results of 16S rRNA gene iTag analysis of toluene-producing enrichment culture

Taxon (genus level)	Relative abundance in community	
k__Bacteria;p__Proteobacteria;c__Gammaproteobacteria;o__Enterobacteriales;f__Enterobacteriaceae;;Other	6.7E-01	i.e., 67%
k__Bacteria;p__Firmicutes;c__Clostridia;o__Clostridiales;f__Veillonellaceae;g__Acidaminococcus;	7.5E-02	
k__Bacteria;p__Proteobacteria;c__Deltaproteobacteria;o__Desulfovibrionales;f__Desulfovibrionaceae;g__Desulfovibrio;	3.9E-02	
k__Archaea;p__Euryarchaeota;c__Methanobacteria;o__Methanobacteriales;f__Methanobacteriaceae;g__Methanobacterium;	3.6E-02	
k__Bacteria;p__Proteobacteria;c__Gammaproteobacteria;o__Enterobacteriales;f__Enterobacteriaceae;g__Kluyvera;	3.0E-02	
k__Bacteria;p__Bacteroidetes;c__Bacteroidia;o__Bacteroidales;;Other;Other	2.9E-02	
k__Bacteria;p__Bacteroidetes;;Other;Other;Other;Other	2.3E-02	
k__Bacteria;p__Actinobacteria;c__Actinobacteria (class);o__Coriobacteriales;f__Coriobacteriaceae;g__Atopobium;	2.2E-02	
k__Bacteria;p__Bacteroidetes;c__Bacteroidia;o__Bacteroidales;f__Prevotellaceae;g__Prevotella;	1.4E-02	
k__Bacteria;p__Tenericutes;c__Erysipelotrichi;o__Erysipelotrichales;f__Erysipelotrichaceae;g__Bulleidia;	1.2E-02	
k__Bacteria;p__Spirochaetes;c__Spirochaetes (class);o__Spirochaetales;f__Spirochaetaceae;g__Treponema;	6.3E-03	
k__Bacteria;p__Tenericutes;c__Erysipelotrichi;o__Erysipelotrichales;f__vadinHA31;g__RFN20;	4.8E-03	
k__Bacteria;p__Firmicutes;c__Clostridia;o__Clostridiales;f__Ruminococcaceae;g__Oscillospira;	4.8E-03	
k__Bacteria;;Other;Other;Other;Other;Other	3.2E-03	
k__Bacteria;p__Bacteroidetes;c__Bacteroidia;o__Bacteroidales;f__Bacteroidaceae;g__Bacteroides;	2.8E-03	
k__Bacteria;p__Firmicutes;c__Clostridia;o__Clostridiales;f__Lachnospiraceae;g__Butyrivibrio;	2.4E-03	
k__Bacteria;p__Proteobacteria;c__Betaproteobacteria;o__Rhodocyclales;f__Rhodocyclaceae;;Other	2.4E-03	
k__Bacteria;p__Firmicutes;c__Clostridia;o__Clostridiales;f__Eubacteriaceae;g__Eubacterium;	2.3E-03	
k__Bacteria;p__Firmicutes;c__Clostridia;o__Clostridiales;;Other;Other	2.3E-03	
k__Bacteria;p__Proteobacteria;c__Gammaproteobacteria;;Other;Other;Other	2.1E-03	
k__Bacteria;p__Firmicutes;c__Clostridia;o__Clostridiales;f__Ruminococcaceae;;Other	2.0E-03	
k__Bacteria;p__Firmicutes;c__Clostridia;o__Clostridiales;f__Veillonellaceae;g__Megasphaera;	1.9E-03	
k__Bacteria;p__Firmicutes;c__Clostridia;o__Clostridiales;f__Lachnospiraceae;;Other	1.1E-03	
k__Bacteria;p__Firmicutes;c__Clostridia;o__Clostridiales;f__Clostridiaceae;g__Clostridium;	9.2E-04	
k__Bacteria;p__Actinobacteria;c__Actinobacteria (class);o__Coriobacteriales;f__Coriobacteriaceae;;Other	8.9E-04	
k__Bacteria;p__Firmicutes;c__Clostridia;o__Clostridiales;f__Veillonellaceae;g__Selenomonas;	7.2E-04	
k__Bacteria;p__Firmicutes;;Other;Other;Other;Other	6.8E-04	
k__Bacteria;p__Firmicutes;c__Clostridia;;Other;Other;Other	6.6E-04	
k__chloro_Populus;p__chloro_Populus;c__chloro_Populus;o__chloro_Populus;f__chloro_Populus;g__chloro_Populus;	6.3E-04	
k__Bacteria;p__Firmicutes;c__Clostridia;o__Clostridiales;f__Ruminococcaceae;g__Ruminococcus;	6.1E-04	
k__Bacteria;p__Firmicutes;c__Clostridia;o__Clostridiales;f__Veillonellaceae;g__Anaeroglobus;	5.6E-04	
k__Bacteria;p__Bacteroidetes;c__Sphingobacteria;o__Sphingobacteriales;;Other;Other	5.6E-04	
k__Bacteria;p__Proteobacteria;c__Epsilonproteobacteria;o__Campylobacterales;f__Campylobacteraceae;g__Sulfurospirillum;	5.5E-04	
k__Bacteria;p__Proteobacteria;;Other;Other;Other;Other	5.4E-04	
k__Bacteria;p__Acidobacteria;c__Acidobacteria (class);o__Acidobacteriales;f__Acidobacteriaceae;g__Terriglobus;	4.6E-04	
k__Bacteria;p__Bacteroidetes;c__Bacteroidia;o__Bacteroidales;f__Porphyromonadaceae;;Other	4.5E-04	
k__Bacteria;p__Firmicutes;c__Clostridia;o__Clostridiales;f__Veillonellaceae;;Other	4.3E-04	
k__Archaea;p__Euryarchaeota;c__Methanomicrobia;o__Methanosarcinales;f__Methanosarcinaceae;g__Methanosarcina;	3.3E-04	
k__Bacteria;p__Firmicutes;c__Clostridia;o__Clostridiales;f__Ruminococcaceae;g__Anaerofilum;	3.1E-04	
k__Bacteria;p__Firmicutes;c__Clostridia;o__Clostridiales;f__Ruminococcaceae;g__Subdoligranulum;	3.1E-04	
k__Bacteria;p__Proteobacteria;c__Deltaproteobacteria;o__Desulfovibrionales;;Other;Other	3.0E-04	
k__Bacteria;p__Firmicutes;c__Clostridia;o__Clostridiales;f__Lachnospiraceae;g__Coprococcus;	2.9E-04	
k__Bacteria;p__Proteobacteria;c__Gammaproteobacteria;o__Pseudomonadales;f__Pseudomonadaceae;g__Pseudomonas;	2.6E-04	
k__Bacteria;p__Proteobacteria;c__Gammaproteobacteria;o__Enterobacteriales;f__Enterobacteriaceae;g__Tatumella;	2.3E-04	
k__Bacteria;p__Firmicutes;c__Clostridia;o__Clostridiales;f__Lachnospiraceae;g__Moryella;	2.2E-04	
k__Bacteria;p__Spirochaetes;c__Spirochaetes (class);o__Spirochaetales;f__Spirochaetaceae;;Other	2.0E-04	
k__Bacteria;p__Proteobacteria;c__Gammaproteobacteria;o__Enterobacteriales;f__Enterobacteriaceae;g__Klebsiella;	2.0E-04	
k__mito_Triticum;p__mito_Triticum;c__mito_Triticum;o__mito_Triticum;f__mito_Triticum;g__mito_Triticum;	1.9E-04	
k__Bacteria;p__Firmicutes;c__Clostridia;o__Clostridiales;f__Veillonellaceae;g__Mitsuokella;	1.3E-04	
k__Bacteria;p__Firmicutes;c__Clostridia;o__Clostridiales;f__Ruminococcaceae;g__Faecalibacterium;	1.3E-04	
k__chloro_Gracilaria;p__chloro_Gracilaria;c__chloro_Gracilaria;o__chloro_Gracilaria;f__chloro_Gracilaria;g__chloro_Gracilaria;	1.2E-04	
k__Bacteria;p__Firmicutes;c__Clostridia;o__Clostridiales;f__Ruminococcaceae;g__Ethanoligenens;	1.2E-04	
k__Bacteria;p__Firmicutes;c__Clostridia;o__Clostridiales;f__Eubacteriaceae;;Other	9.7E-05	
k__Bacteria;p__Bacteroidetes;c__Bacteroidia;o__Bacteroidales;f__Porphyromonadaceae;g__Parabacteroides;	7.4E-05	
k__Archaea;p__Euryarchaeota;;Other;Other;Other;Other	6.3E-05	
k__Bacteria;p__Actinobacteria;c__Actinobacteria (class);o__Coriobacteriales;f__Coriobacteriaceae;g__Coriobacterium;	4.3E-05	
k__Archaea;p__Euryarchaeota;c__Methanomicrobia;o__Methanomicrobiales;f__Methanomicrobiaceae;g__Methanofollis;	3.6E-05	

k__Bacteria;p__Bacteroidetes;c__Bacteroidia;o__Bacteroidales;f__Porphyromonadaceae;g__Candidatus Azobacteroides;	2.9E-05
k__Bacteria;p__Chloroflexi;c__Anaerolineae;o__Anaerolineales;f__Anaerolinaceae;g__WCHB1-05;	2.4E-05
k__Bacteria;p__Firmicutes;c__Clostridia;o__Clostridiales;f__Ruminococcaceae;g__Anaerotruncus;	2.3E-05
k__Bacteria;p__Proteobacteria;c__Deltaproteobacteria;;Other;Other;Other	2.0E-05
k__Bacteria;p__Proteobacteria;c__Gammaproteobacteria;o__Alteromonadales;f__Alteromonadaceae;;Other	1.9E-05
k__Bacteria;p__Firmicutes;c__Clostridia;o__Clostridiales;f__Dehalobacteriaceae;g__Dehalobacterium;	1.8E-05
k__Bacteria;p__Proteobacteria;c__Betaproteobacteria;;Other;Other;Other	1.7E-05
k__Bacteria;p__Firmicutes;c__Bacilli;o__Haloplasmatales;f__Haloplasmataceae;g__Haloplasma;	1.7E-05
k__Bacteria;p__Acidobacteria;c__Acidobacteria (class);o__Acidobacteriales;f__Acidobacteriaceae;;Other	1.7E-05
k__Bacteria;p__Proteobacteria;c__Gammaproteobacteria;o__Enterobacteriales;f__Enterobacteriaceae;g__Trabulsiella;	1.6E-05
k__Bacteria;p__Firmicutes;c__Clostridia;o__Natranaerobiales;f__Anaerobrancaceae;;Other	1.6E-05
k__Bacteria;p__Bacteroidetes;c__Flavobacteria;o__Flavobacteriales;f__Flavobacteriaceae;g__Flavobacterium;	1.4E-05
k__Bacteria;p__Firmicutes;c__Clostridia;o__Natranaerobiales;f__Anaerobrancaceae;g__KF-Gitt2-16;	1.3E-05
k__Archaea;p__Euryarchaeota;c__Methanobacteria;o__Methanobacteriales;f__Methanobacteriaceae;g__Methanosphaera;	1.3E-05
k__mito_Bambusa;p__mito_Bambusa;c__mito_Bambusa;o__mito_Bambusa;f__mito_Bambusa;g__mito_Bambusa;	1.2E-05
k__Bacteria;p__Proteobacteria;c__Deltaproteobacteria;o__Desulfovibrionales;f__Desulfovibrionaceae;;Other	1.2E-05
k__Bacteria;p__Proteobacteria;c__Alphaproteobacteria;o__Rhizobiales;f__Rhizobiaceae;g__Rhizobium;	1.2E-05
k__Bacteria;p__Firmicutes;c__Clostridia;o__Clostridiales;f__Lachnospiraceae;g__Anaerostipes;	1.2E-05
k__Bacteria;p__Firmicutes;c__Clostridia;o__Clostridiales;f__Eubacteriaceae;g__Pseudoramibacter;	1.2E-05
k__mito_Megaceros;p__mito_Megaceros;c__mito_Megaceros;o__mito_Megaceros;f__mito_Megaceros;g__mito_Megaceros;	9.6E-06
k__Bacteria;p__Proteobacteria;c__Alphaproteobacteria;o__Rhizobiales;f__Bradyrhizobiaceae;g__Rhodopseudomonas;	9.6E-06
k__Bacteria;p__Actinobacteria;c__Actinobacteria (class);o__Actinomycetales;f__Streptomycetaceae;g__Streptomyces;	9.6E-06
k__Bacteria;p__Acidobacteria;;Other;Other;Other;Other	9.6E-06
k__Bacteria;p__Tenericutes;c__Erysipelotrichi;o__Erysipelotrichales;f__Erysipelotrichaceae;;Other	8.4E-06
k__Bacteria;p__Tenericutes;c__Erysipelotrichi;o__Erysipelotrichales;;Other;Other	8.4E-06
k__Bacteria;p__Proteobacteria;c__Betaproteobacteria;o__Burkholderiales;f__Comamonadaceae;;Other	8.4E-06
k__Bacteria;p__Firmicutes;c__Clostridia;o__Clostridiales;f__Veillonellaceae;g__Thermosinus;	8.4E-06
k__Bacteria;p__Chlorobi;c__Ignavibacteria;o__Ignavibacteriales;f__Ignavibacteriaceae;g__Ignavibacterium;	8.4E-06
k__Bacteria;p__Proteobacteria;c__Gammaproteobacteria;o__Pseudomonadales;f__Moraxellaceae;g__Acinetobacter;	7.2E-06
k__Bacteria;p__Proteobacteria;c__Gammaproteobacteria;o__Enterobacteriales;f__Enterobacteriaceae;g__Cedecea;	7.2E-06
k__Bacteria;p__Proteobacteria;c__Deltaproteobacteria;o__Desulfovibrionales;f__Desulfohalobiaceae;;Other	7.2E-06
k__Bacteria;p__Synergistetes;c__Synergistia;o__Synergistales;f__Synergistaceae;g__Candidatus Tammella;	6.0E-06
k__Bacteria;p__Proteobacteria;c__Gammaproteobacteria;o__Enterobacteriales;f__Enterobacteriaceae;g__Shigella;	6.0E-06
k__Bacteria;p__Proteobacteria;c__Alphaproteobacteria;o__Rhizobiales;f__Rhizobiaceae;g__Agrobacterium;	6.0E-06
k__Bacteria;p__Firmicutes;c__Clostridia;o__Clostridiales;f__Eubacteriaceae;g__Acetobacterium;	6.0E-06
k__Bacteria;p__Chloroflexi;c__Anaerolineae;o__Anaerolineales;f__Anaerolinaceae;;Other	6.0E-06
k__Bacteria;p__Actinobacteria;c__Actinobacteria (class);;Other;Other;Other	6.0E-06
k__Bacteria;p__Proteobacteria;c__Betaproteobacteria;o__Burkholderiales;f__Comamonadaceae;g__Variovorax;	4.8E-06
k__Bacteria;p__Bacteroidetes;c__Sphingobacteria;o__Sphingobacteriales;f__Flammeovirgaceae;;Other	4.8E-06
k__Bacteria;p__Bacteroidetes;c__Flavobacteria;o__Flavobacteriales;f__Flavobacteriaceae;g__Chryseobacterium;	4.8E-06
k__Archaea;p__Euryarchaeota;c__Methanobacteria;o__Methanobacteriales;f__Methanobacteriaceae;g__Methanobrevibacter;	4.8E-06
k__chloro_Nicotiana;p__chloro_Nicotiana;c__chloro_Nicotiana;o__chloro_Nicotiana;f__chloro_Nicotiana;g__chloro_Nicotiana;	3.6E-06
k__chloro_Chlorella;p__chloro_Chlorella;c__chloro_Chlorella;o__chloro_Chlorella;f__chloro_Chlorella;g__chloro_Chlorella;	3.6E-06
k__Bacteria;p__Synergistetes;c__Synergistia;o__Synergistales;f__Synergistaceae;g__Synergistes;	3.6E-06
k__Bacteria;p__Proteobacteria;c__Gammaproteobacteria;o__Enterobacteriales;f__Enterobacteriaceae;g__Averyella;	3.6E-06
k__Bacteria;p__Proteobacteria;c__Alphaproteobacteria;o__Caulobacterales;f__Caulobacteraceae;g__Phenylobacterium;	3.6E-06
k__Bacteria;p__Actinobacteria;c__Actinobacteria (class);o__Actinomycetales;f__Streptosporangiaceae;g__Nonomuraea;	3.6E-06
k__mito_Zea;p__mito_Zea;c__mito_Zea;o__mito_Zea;f__mito_Zea;g__mito_Zea;	2.4E-06
k__mito_Nicotiana;p__mito_Nicotiana;c__mito_Nicotiana;o__mito_Nicotiana;f__mito_Nicotiana;g__mito_Nicotiana;	2.4E-06
k__Metazoa;;Other;Other;Other;Other;Other	2.4E-06
k__Bacteria;p__Proteobacteria;c__Gammaproteobacteria;o__Xanthomonadales;f__Xanthomonadaceae;g__Stenotrophomonas;	2.4E-06
k__Bacteria;p__Proteobacteria;c__Gammaproteobacteria;o__Enterobacteriales;f__Enterobacteriaceae;g__Yersinia;	2.4E-06
k__Bacteria;p__Proteobacteria;c__Gammaproteobacteria;o__Enterobacteriales;f__Enterobacteriaceae;g__Salmonella;	2.4E-06
k__Bacteria;p__Proteobacteria;c__Betaproteobacteria;o__Burkholderiales;f__Oxalobacteraceae;g__Massilia;	2.4E-06
k__Bacteria;p__Proteobacteria;c__Alphaproteobacteria;o__Sphingomonadales;f__Sphingomonadaceae;;Other	2.4E-06
k__Bacteria;p__Proteobacteria;c__Alphaproteobacteria;o__Rhizobiales;f__Hyphomicrobiaceae;g__Rhodoplanes;	2.4E-06
k__Bacteria;p__Firmicutes;c__Clostridia;o__Clostridiales;f__Lachnospiraceae;g__Johnsonella;	2.4E-06
k__Bacteria;p__Firmicutes;c__Bacilli;;Other;Other;Other	2.4E-06
k__Bacteria;p__Bacteroidetes;c__Flavobacteria;o__Flavobacteriales;f__Flavobacteriaceae;;Other	2.4E-06
k__Archaea;;Other;Other;Other;Other;Other	2.4E-06

Zargar, Saville, Phelan, Tringe, Petzold, Keasling, and Beller. *In vitro* Characterization of Phenylacetate Decarboxylase, a Novel Enzyme Catalyzing Toluene Biosynthesis in an Anaerobic Microbial Community, *Scientific Reports*

Table S2. Shotgun proteomic results for FPLC fractions 18 (inactive) and 19 (active) in Figure 2 [results only shown for proteins having greater total unique peptides in active fraction]

* Total unique peptides

Ranked according to F19 - F18

Locus tag	F18 (inactive)*	F19 (active)*	F19-F18 diff.*	COG	COG alpha	EC	KO	Pfam	Best JGI annotation
JGI20225J20221_100038726	149	221	72	4058	H	2.8.4.1	K00399	2745	Methyl coenzyme M reductase, alpha subunit
JGI20225J20221_100038725	89	147	58	4057	H	2.8.4.1	K00402	2240	Methyl coenzyme M reductase, gamma subunit
JGI20225J20221_10000011633	23	63	40	191	G	4.1.2.13	K01624	1116	Fructose/tagatose bisphosphate aldolase
JGI20225J20221_10000011513	81	118	37	148	G	4.2.1.11	K01689	113	Enolase
JGI20225J20221_100050178	6	42	36	282	C	2.7.2.1	K00925	871	Acetate kinase
JGI20225J20221_100038722	81	115	34	4054	H	2.8.4.1	K00401	2241	Methyl coenzyme M reductase, beta subunit
JGI20225J20221_10008678	31	63	32	4624	C	1.6.5.3	K00336	2906	Iron only hydrogenase large subunit, C-terminal domain
JGI20225J20221_1000001608	28	56	28	1882	C	2.3.1.54	K00656	2901	Pyruvate-formate lyase
JGI20225J20221_10000011884	12	36	24	137	E	6.3.4.5	K01940	764	Argininosuccinate synthase
JGI20225J20221_1000250202	20	41	21	4624	C	1.6.5.3	K00336	2906	Iron only hydrogenase large subunit, C-terminal domain
JGI20225J20221_10005705	13	34	21	22	C	1.2.4.1	K00162	2779	Pyruvate/2-oxoglutarate dehydrogenase complex, dehydrogenase (E1) component, eukaryotic type, beta subunit
JGI20225J20221_1000079825	21	39	18	57	G	1.2.1.12	K00134	44	Glyceraldehyde-3-phosphate dehydrogenase/erythrose-4-phosphate dehydrogenase
JGI20225J20221_1000014106	10	28	18	362	G	1.1.1.44	K00033	393	6-phosphogluconate dehydrogenase
JGI20225J20221_1000084518	14	30	16	1866	C	4.1.1.49	K01610	1293	Phosphoenolpyruvate carboxykinase (ATP)
JGI20225J20221_1000004958	2	18	16	1148	C	1.8.98.1	K03388	7992	Heterodisulfide reductase, subunit A and related polyferredoxins
JGI20225J20221_100002653	0	15	15	1960	I	1.3.8.1	K00248	441	Acyl-CoA dehydrogenases
JGI20225J20221_10000041265	0	13	13	3259	C	1.12.99.-	K14126	374	Coenzyme F420-reducing hydrogenase, alpha subunit
JGI20225J20221_10000012039	22	35	13	50	J		K02358	9	GTPases - translation elongation factors
JGI20225J20221_10000011162	0	13	13	450	V	1.11.1.15	K03386	578	Peroxiredoxin
JGI20225J20221_10005704	0	12	12	1071	C	1.2.4.1	K00161	676	Pyruvate/2-oxoglutarate dehydrogenase complex, dehydrogenase (E1) component, eukaryotic type, alpha subunit
JGI20225J20221_10000011627	17	29	12	111	HR	1.1.1.95	K00058	2826	Phosphoglycerate dehydrogenase and related dehydrogenases
JGI20225J20221_1000004820	42	53	11	1927	C	1.5.99.9	K00319	1993	Coenzyme F420-dependent N(5),N(10)-methenyltetrahydromethanopterin dehydrogenase
JGI20225J20221_100066655	0	11	11	111	HR	1.1.1.95	K00058	2826	Phosphoglycerate dehydrogenase and related dehydrogenases
JGI20225J20221_10008677	12	22	10	1894	C	1.6.5.3	K00335	1512	NADH:ubiquinone oxidoreductase, NADH-binding (51 kD) subunit
JGI20225J20221_10187692	0	10	10	1960	I		K00257	441	Acyl-CoA dehydrogenases
JGI20225J20221_100251813	0	10	10	1960	I		K00257	441	Acyl-CoA dehydrogenases
JGI20225J20221_10221552	0	9	9	1454	C	1.1.1.1	K13954	465	Alcohol dehydrogenase, class IV
JGI20225J20221_1000001216	0	9	9	171	H	6.3.1.5	K01916	2540	NAD synthase
JGI20225J20221_100002933	23	32	9	264	J		K02357	889	Translation elongation factor Ts
JGI20225J20221_100174214	2	11	9			2.1.1.90	K04480	12176	Methanol-cobalamin methyltransferase B subunit
JGI20225J20221_100035070	3	11	8	2352	C	4.1.1.31	K01595	311	Phosphoenolpyruvate carboxylase
JGI20225J20221_100029386	5	13	8	22	C	1.2.4.1	K00162	2779	Pyruvate/2-oxoglutarate dehydrogenase complex, dehydrogenase (E1) component, eukaryotic type, beta subunit
JGI20225J20221_100047250	22	30	8	574	G	2.7.9.1	K01006	2896	Phosphoenolpyruvate synthase/pyruvate phosphate dikinase
JGI20225J20221_100111320	16	24	8	191	G	4.1.2.13	K01624	1116	Fructose/tagatose bisphosphate aldolase
JGI20225J20221_1000014397	12	20	8	2190	G	2.7.1.69	K02777	358	Phosphotransferase system IIA components
JGI20225J20221_1000029176	19	27	8	443	O		K04043	12	Molecular chaperone
JGI20225J20221_10000011125	0	8	8	544	O		K03545	5697	FKBP-type peptidyl-prolyl cis-trans isomerase (trigger factor)
JGI20225J20221_10044662	5	12	7	1529	C			2738	Aerobic-type carbon monoxide dehydrogenase, large subunit CoxL/CutL homologs
JGI20225J20221_1000079191	2	9	7	674	C	1.2.7.-	K03737	1855	Pyruvate:ferredoxin oxidoreductase and related 2-oxoacid:ferredoxin oxidoreductases, alpha subunit
JGI20225J20221_1000084466	0	7	7	674	C	1.2.7.-	K03737	1855	Pyruvate:ferredoxin oxidoreductase and related 2-oxoacid:ferredoxin oxidoreductases, alpha subunit
JGI20225J20221_1000014451	2	9	7	150	F	6.3.3.1	K01933	2769	Phosphoribosylaminoimidazole (AIR) synthetase
JGI20225J20221_1000014762	0	7	7	696	G	5.4.2.1	K15633	1676	Phosphoglyceromutase
JGI20225J20221_10000041512	34	41	7	2141	HR	1.5.99.11	K00320	296	Coenzyme F420-dependent N5,N10-methylene tetrahydromethanopterin reductase and related flavin-dependent oxidoreductases
JGI20225J20221_1000017329	0	7	7	1210	M	2.7.7.9	K00963	483	UDP-glucose pyrophosphorylase
JGI20225J20221_10220831	3	9	6	674	C	1.2.7.-	K03737	1855	Pyruvate:ferredoxin oxidoreductase and related 2-oxoacid:ferredoxin oxidoreductases, alpha subunit
JGI20225J20221_10006669	7	13	6	655	C			3358	Multimeric flavodoxin WrbA
JGI20225J20221_1000014311	5	11	6	282	C	2.7.2.1	K00925	871	Acetate kinase
JGI20225J20221_1000014640	3	9	6	55	C	3.6.3.14	K02112	6	F0F1-type ATP synthase, beta subunit
JGI20225J20221_1000011547	1	7	6	1150	C	1.8.98.1	K03390	13183	Heterodisulfide reductase, subunit C
JGI20225J20221_100126347	0	6	6	55	C	3.6.3.14	K02112	6	F0F1-type ATP synthase, beta subunit
JGI20225J20221_10058031	0	6	6	1866	C	4.1.1.49	K01610	1293	Phosphoenolpyruvate carboxykinase (ATP)
JGI20225J20221_1000011685	0	6	6	221	CP	3.6.1.1	K01507	719	Inorganic pyrophosphatase
JGI20225J20221_10000012148	17	23	6	683	E		K01999	13458	ABC-type branched-chain amino acid transport systems, periplasmic component
JGI20225J20221_10000011636	9	15	6	21	G	2.2.1.1	K00615	456	Transketolase
JGI20225J20221_1000001180	3	9	6	574	G	2.7.9.2	K01007	1326	Phosphoenolpyruvate synthase/pyruvate phosphate dikinase
JGI20225J20221_1000001826	0	6	6	588	G	5.4.2.1	K01834	300	Phosphoglycerate mutase 1
JGI20225J20221_1000084443	0	6	6	448	G	2.7.7.27	K00975	483	ADP-glucose pyrophosphorylase
JGI20225J20221_100043370	0	6	6	1812	H	2.5.1.6	K00789	1941	Archaeal S-adenosylmethionine synthetase
JGI20225J20221_10000011052	0	6	6	652	O	5.2.1.8	K03768	160	Peptidyl-prolyl cis-trans isomerase (rotamase) - cyclophilin family
JGI20225J20221_10000012202	0	6	6	589	T		K06149	582	Universal stress protein UspA and related nucleotide-binding proteins
JGI20225J20221_1000001308	33	38	5	538	C	1.1.1.42	K00031	180	Isocitrate dehydrogenases
JGI20225J20221_1000250203	12	17	5	1894	C	1.6.5.3	K00335	1512	NADH:ubiquinone oxidoreductase, NADH-binding (51 kD) subunit
JGI20225J20221_100115946	0	5	5	1529	C			2738	Aerobic-type carbon monoxide dehydrogenase, large subunit CoxL/CutL homologs
JGI20225J20221_1000014638	5	10	5	56	C	3.6.3.14	K02111	6	F0F1-type ATP synthase, alpha subunit
JGI20225J20221_10000041335	2	7	5	1155	C	3.6.3.14	K02117	6	Archaeal/vacuolar-type H+-ATPase subunit A
JGI20225J20221_100002655	0	5	5	2025	C		K03522	1012	Electron transfer flavoprotein, alpha subunit
JGI20225J20221_10221481	0	5	5	1042	C		K09181	13549	Acyl-CoA synthetase (NDP forming)
JGI20225J20221_100042415	0	5	5	1394	C	3.6.3.14	K02120	1813	Archaeal/vacuolar-type H+-ATPase subunit D
JGI20225J20221_10000011949	0	5	5	39	C	1.1.1.37	K00024	56	Malate/lactate dehydrogenases
JGI20225J20221_1000061151	0	5	5	674	C	1.2.7.3	K00174	1855	Pyruvate:ferredoxin oxidoreductase and related 2-oxoacid:ferredoxin oxidoreductases, alpha subunit

Locus tag	F18 (inactive)*	F19 (active)*	F19-F18 diff.*	COG	COG alpha	EC	KO	Pfam	Best JGI annotation
JGI20225J20221_1000051340	0	5	5	55	C	3.6.3.14	K02112	6	F0F1-type ATP synthase, beta subunit
JGI20225J20221_100035014	31	36	5	174	E	6.3.1.2	K01915	120	Glutamine synthetase
JGI20225J20221_1000001645	3	8	5	2008	E	4.1.2.5	K01620	1212	Threonine aldolase
JGI20225J20221_1000011170	0	5	5	1363	EG	3.2.1.4	K01179	5343	Cellulase M and related proteins
JGI20225J20221_1000026272	4	9	5	46	F	6.3.5.3	K01952	13507	Phosphoribosylformylglycinamidine (FGAM) synthase, synthetase domain
JGI20225J20221_1000015522	15	20	5	191	G	4.1.2.13	K01624	1116	Fructose/tagatose bisphosphate aldolase
JGI20225J20221_100041045	0	5	5	422	H		K03147	1964	Thiamine biosynthesis protein ThiC
JGI20225J20221_1000014870	0	5	5	776	L		K05787	216	Bacterial nucleoid DNA-binding protein
JGI20225J20221_1000001170	14	19	5	3383	R	1.2.1.2	K00123	384	Uncharacterized anaerobic dehydrogenase
JGI20225J20221_1000001457	13	17	4	655	C		K03809	3358	Multimeric flavodoxin WrbA
JGI20225J20221_100115947	2	6	4	1529	C			2738	Aerobic-type carbon monoxide dehydrogenase, large subunit CoxL/CutL homologs
JGI20225J20221_100085226	6	10	4	1592	C			2915	Rubrerythrin
JGI20225J20221_100042414	4	8	4	1156	C	3.6.3.14	K02118	6	Archaeal/vacuolar-type H+-ATPase subunit B
JGI20225J20221_10072021	0	4	4	1042	C		K09181	13607	Acyl-CoA synthetase (NDP forming)
JGI20225J20221_100057013	0	4	4	1249	C	1.8.1.4	K00382	7992	Pyruvate/2-oxoglutarate dehydrogenase complex, dihydrolipoamide dehydrogenase (E3) component, and related enzymes
JGI20225J20221_100089039	1	5	4	5012	C		K14081	2310	Predicted cobalamin binding protein - COG5012
JGI20225J20221_10000041264	0	4	4	1941	C	1.12.99.-	K14128	1058	Coenzyme F420-reducing hydrogenase, gamma subunit
JGI20225J20221_1000017326	0	4	4	1454	C	1.1.1.1	K04072	465	Alcohol dehydrogenase, class IV
JGI20225J20221_100058762	0	4	4	1227	CP	3.6.1.1	K01507	2833	Inorganic pyrophosphatase/exopolyphosphatase
JGI20225J20221_10000011968	0	4	4	604	CR			107	NADPH:quinone reductase and related Zn-dependent oxidoreductases
JGI20225J20221_10058163	6	10	4	69	E			1645	Glutamate synthase domain 2
JGI20225J20221_100036175	47	51	4	59	EH	1.1.1.86	K00053	7991	Ketol-acid reductoisomerase
JGI20225J20221_1000051484	2	6	4	1146	F	1.8.99.2	K00395	12139	Ferredoxin
JGI20225J20221_10052911	6	10	4	57	G			44	Glyceraldehyde-3-phosphate dehydrogenase/erythrose-4-phosphate dehydrogenase
JGI20225J20221_1000061295	8	12	4	149	G	5.3.1.1	K01803	121	Triosephosphate isomerase
JGI20225J20221_10771093	1	5	4	469	G	2.7.1.40	K00873	224	Pyruvate kinase
JGI20225J20221_100035042	0	4	4	205	G	2.7.1.11	K00850	365	6-phosphofructokinase
JGI20225J20221_10000041244	0	4	4	4058	H	2.8.4.1	K00399	2745	Methyl coenzyme M reductase, alpha subunit
JGI20225J20221_100047261	1	5	4	264	J		K02357	889	Translation elongation factor Ts
JGI20225J20221_1000038248	0	4	4	264	J		K02357	889	Translation elongation factor Ts
JGI20225J20221_1000001616	0	4	4	172	J	6.1.1.11	K01875	587	Seryl-tRNA synthetase
JGI20225J20221_100138824	0	4	4	480	J		K02355	9	Translation elongation factors (GTPases)
JGI20225J20221_1000014404	0	4	4	2837	P		K07223	4261	Predicted iron-dependent peroxidase - COG2837
JGI20225J20221_100002979	7	10	3	1049	C	4.2.1.3	K01682	6434	Aconitase B
JGI20225J20221_100000472	4	7	3	1592	C			2915	Rubrerythrin
JGI20225J20221_100050136	0	3	3	1740	C	1.12.2.-	K14070	1058	Ni,Fe-hydrogenase I small subunit
JGI20225J20221_1000018318	0	3	3	674	C	1.2.7.-	K03737	1855	Pyruvate:ferredoxin oxidoreductase and related 2-oxoacid:ferredoxin oxidoreductases, alpha subunit
JGI20225J20221_1000018772	0	3	3	4624	C	1.6.5.3	K00336	2906	Iron only hydrogenase large subunit, C-terminal domain
JGI20225J20221_10068971	0	3	3	2025	C		K03522	1012	Electron transfer flavoprotein, alpha subunit
JGI20225J20221_100002937	3	6	3	2171	E	2.3.1.117	K00674	14805	Tetrahydrodipicolinate N-succinyltransferase
JGI20225J20221_1000017299	0	3	3	159	E	4.2.1.20	K01695	290	Tryptophan synthase alpha chain
JGI20225J20221_1000026357	0	3	3	137	E	6.3.4.5	K01940	764	Argininosuccinate synthase
JGI20225J20221_100036179	5	8	3	115	EH	2.6.1.42	K00826	1063	Branched-chain amino acid aminotransferase/4-amino-4-deoxychorismate lyase
JGI20225J20221_100006145	1	4	3	104	F	6.3.4.4	K01939	709	Adenylosuccinate synthase
JGI20225J20221_1000001313	0	3	3	15	F	4.3.2.2	K01756	206	Adenylosuccinate lyase
JGI20225J20221_1000014531	0	3	3	519	F	6.3.5.2	K01951	958	GMP synthase, PP-ATPase domain/subunit
JGI20225J20221_1000165127	0	3	3	2759	F	6.3.4.3	K01938	1268	Formyltetrahydrofolate synthetase
JGI20225J20221_10019235	16	19	3	1080	G	2.7.3.9	K08483	2896	Phosphoenolpyruvate-protein kinase (PTS system EI component in bacteria)
JGI20225J20221_1000045267	8	11	3	1080	G	2.7.3.9	K08483	2896	Phosphoenolpyruvate-protein kinase (PTS system EI component in bacteria)
JGI20225J20221_1000017472	1	4	3	469	G	2.7.1.40	K00873	224	Pyruvate kinase
JGI20225J20221_1000031379	0	3	3	1109	G	5.4.2.2	K01835	2878	Phosphomannomutase
JGI20225J20221_100006412	0	3	3	469	G	2.7.1.40	K00873	224	Pyruvate kinase
JGI20225J20221_10000011149	6	9	3	54	H	2.5.1.78	K00794	885	Riboflavin synthase beta-chain
JGI20225J20221_1000004949	0	3	3	1812	H	2.5.1.6	K00789	1941	Archaeal S-adenosylmethionine synthetase
JGI20225J20221_10016373	1	4	3	214	H	4.-	K06215	1680	Pyridoxine biosynthesis enzyme
JGI20225J20221_10000011973	0	3	3	439	I	6.3.4.14	K01961	2786	Biotin carboxylase
JGI20225J20221_1000014343	0	3	3	304	IQ	2.3.1.41	K00647	109	3-oxoacyl-(acyl-carrier-protein) synthase
JGI20225J20221_100029383	0	3	3	1028	IQR	1.1.1.303	K03366	106	Dehydrogenases with different specificities (related to short-chain alcohol dehydrogenases)
JGI20225J20221_1000079362	0	3	3	50	J		K02358	9	GTPases - translation elongation factors
JGI20225J20221_1000061329	0	3	3	480	J		K02355	3764	Translation elongation factors (GTPases)
JGI20225J20221_10000011928	2	5	3	525	J	6.1.1.9	K01873	133	Valyl-tRNA synthetase
JGI20225J20221_1000243321	0	3	3	2092	J		K03232	736	Translation elongation factor EF-1beta
JGI20225J20221_1000001602	0	3	3	539	J		K02945	575	Ribosomal protein S1
JGI20225J20221_1000017358	2	5	3	2877	M	2.5.1.55	K01627	793	3-deoxy-D-manno-octulosonic acid (KDO) 8-phosphate synthase
JGI20225J20221_100051271	0	3	3	459	O			118	Chaperonin GroEL (HSP60 family)
JGI20225J20221_1000243313	0	3	3	443	O		K04043	12	Molecular chaperone
JGI20225J20221_100047876	0	3	3	459	O		K04077	118	Chaperonin GroEL (HSP60 family)
JGI20225J20221_100073661	0	3	3	535	R		K02585	4055	Predicted Fe-S oxidoreductases - COG0535
JGI20225J20221_1000185100	0	3	3			2.1.1.90	K04480	12176	Methanol-cobalamin methyltransferase B subunit
JGI20225J20221_100166348	0	3	3						
JGI20225J20221_1000189347	8	10	2	1866	C	4.1.1.49	K01610	1293	Phosphoenolpyruvate carboxykinase (ATP)
JGI20225J20221_1000177177	6	8	2	1866	C	4.1.1.49	K01610	1293	Phosphoenolpyruvate carboxykinase (ATP)
JGI20225J20221_1000250204	9	11	2	3411	C			7845	Ferredoxin
JGI20225J20221_100035425	0	2	2	1529	C			2738	Aerobic-type carbon monoxide dehydrogenase, large subunit CoxL/CutL homologs
JGI20225J20221_1000638148	0	2	2	674	C	1.2.7.-	K03737	1855	Pyruvate:ferredoxin oxidoreductase and related 2-oxoacid:ferredoxin oxidoreductases, alpha subunit
JGI20225J20221_100089128	8	10	2	4231	C	1.8.99.2	K00395	12139	Adenosine-5'-phosphosulfate reductase beta subunit;
JGI20225J20221_100047923	5	7	2	1592	C			2915	Rubrerythrin

Locus tag	F18 (inactive)*	F19 (active)*	F19-F18 diff.*	COG	COG alpha	EC	KO	Pfam	Best JGI annotation
JGI20225J20221_100029387	0	2	2	1071	C	1.2.4.1	K00161	676	Pyruvate/2-oxoglutarate dehydrogenase complex, dehydrogenase (E1) component, eukaryotic type, alpha subunit
JGI20225J20221_100210616	0	2	2	280	C	2.3.1.8	K00625	1515	Phosphotransacetylase
JGI20225J20221_1000001238	0	2	2	778	C			881	Nitroreductase
JGI20225J20221_100042410	0	2	2	1390	C	3.6.3.14	K02121	1991	Archaeal/vacuolar-type H+-ATPase subunit E
JGI20225J20221_10068972	0	2	2	2086	C		K03521	1012	Electron transfer flavoprotein, beta subunit
JGI20225J20221_1000029366	0	2	2	221	CP	3.6.1.1	K01507	719	Inorganic pyrophosphatase
JGI20225J20221_1000014569	8	10	2	112	E	2.1.2.1	K00600	464	Glycine/serine hydroxymethyltransferase
JGI20225J20221_100001496	2	4	2	79	E	2.6.1.9	K00817	155	Histidinol-phosphate/aromatic aminotransferase and cobyric acid decarboxylase
JGI20225J20221_1000031265	0	2	2	1748	E	1.5.1.7	K00290	3435	Saccharopine dehydrogenase and related proteins
JGI20225J20221_10003615	0	2	2	6	E	3.4.13.9	K01271	557	Xaa-Pro aminopeptidase
JGI20225J20221_1000306123	0	2	2	59	EH	1.1.1.86	K00053	7991	Ketol-acid reductoisomerase
JGI20225J20221_1000034169	0	2	2	150	F	6.3.3.1	K01933	2769	Phosphoribosylaminoimidazole (AIR) synthetase
JGI20225J20221_1000029409	1	3	2	104	F	6.3.4.4	K01939	709	Adenylosuccinate synthase
JGI20225J20221_1000029206	0	2	2	813	F	2.4.2.1	K03784	1048	Purine-nucleoside phosphorylase
JGI20225J20221_1000045144	0	2	2	813	F	2.4.2.1	K03784	1048	Purine-nucleoside phosphorylase
JGI20225J20221_1000026392	0	2	2	528	F	2.7.4.22	K09903	696	Uridylate kinase
JGI20225J20221_1000079249	1	3	2	448	G	2.7.7.27	K00975	483	ADP-glucose pyrophosphorylase
JGI20225J20221_1000029207	0	2	2	1015	G	5.4.2.7	K01839	1676	Phosphopentomutase
JGI20225J20221_1000001161	0	2	2	469	G	2.7.1.40	K00873	224	Pyruvate kinase
JGI20225J20221_1000061256	0	2	2	1105	G	2.7.1.56	K00882	294	Fructose-1-phosphate kinase and related fructose-6-phosphate kinase (PfkB)
JGI20225J20221_10772375	0	2	2	1488	H	2.4.2.11	K00763	4095	Nicotinic acid phosphoribosyltransferase
JGI20225J20221_1000014665	0	2	2	561	HR		K07024	8282	Predicted hydrolases of the HAD superfamily - COG0561
JGI20225J20221_1000045407	2	4	2	264	J		K02357	889	Translation elongation factor Ts
JGI20225J20221_100054713	0	2	2	5256	J		K03231	9	Translation elongation factor EF-1alpha (GTPase)
JGI20225J20221_100006430	0	2	2	143	J	6.1.1.10	K01874	9334	Methionyl-tRNA synthetase
JGI20225J20221_100002935	0	2	2	24	J	3.4.11.18	K01265	557	Methionine aminopeptidase
JGI20225J20221_10000011440	0	2	2	13	J	6.1.1.7	K01872	1411	Alanyl-tRNA synthetase
JGI20225J20221_1000001902	0	2	2	8	J	6.1.1.18	K01886	749	Glutamyl- and glutaminyl-tRNA synthetases
JGI20225J20221_10000011892	1	3	2	782	K		K03624	3449	Transcription elongation factor
JGI20225J20221_100111342	1	3	2	1846	K			1047	Transcriptional regulators
JGI20225J20221_1000004222	0	2	2	2101	K		K03120	352	TATA-box binding protein (TBP), component of TFIID and TFIIIB
JGI20225J20221_10000011902	0	2	2	766	M	2.5.1.7	K00790	275	UDP-N-acetylglucosamine enolpyruvyl transferase
JGI20225J20221_1000001564	0	2	2	2885	M		K03286	1389	Outer membrane protein and related peptidoglycan-associated (lipo)proteins
JGI20225J20221_10001652	0	2	2	835	NT		K03415	1584	Chemotaxis signal transduction protein
JGI20225J20221_1000029447	5	7	2	234	O		K04078	166	Co-chaperonin GroES (HSP10)
JGI20225J20221_10028576	2	4	2	443	O		K04043	12	Molecular chaperone
JGI20225J20221_100087633	0	2	2	459	O		K04077	118	Chaperonin GroEL (HSP60 family)
JGI20225J20221_1000001622	0	2	2	492	O	1.8.1.9	K00384	7992	Thioredoxin reductase
JGI20225J20221_1000186110	0	2	2	1047	O	5.2.1.8	K03775	254	FKBP-type peptidyl-prolyl cis-trans isomerases 2
JGI20225J20221_100105335	0	2	2	1528	P	1.16.3.1	K02217	210	Ferritin-like protein
JGI20225J20221_100016876	0	2	2	2046	P	2.7.7.4	K00958	1747	ATP sulfurylase (sulfate adenylyltransferase)
JGI20225J20221_1000084512	0	2	2	1040	R			12773	Predicted am
JGI20225J20221_100086417	1	3	2	2333	R				
JGI20225J20221_1000315251	0	2	2	589	T			582	Universal stress protein UspA and related nucleotide-binding proteins
JGI20225J20221_1000165125	0	2	2						
JGI20225J20221_10008676	15	16	1	3411	C			7845	Ferredoxin
JGI20225J20221_1000026344	15	16	1	1454	C			465	Alcohol dehydrogenase, class IV
JGI20225J20221_100050177	9	10	1	280	C	2.3.1.8	K00625	1515	Phosphotransacetylase
JGI20225J20221_100229915	0	1	1	56	C			6	F0F1-type ATP synthase, alpha subunit
JGI20225J20221_100017438	0	1	1	1148	C			13187	
JGI20225J20221_100111335	0	1	1	2326	C			3976	Uncharacterized conserved protein - COG2326
JGI20225J20221_1000061152	1	2	1	1013	C	1.2.7.3	K00175	2775	Pyruvate:ferredoxin oxidoreductase and related 2-oxoacid:ferredoxin oxidoreductases, beta subunit
JGI20225J20221_1000243301	0	1	1	644	C			1494	Dehydrogenases (flavoproteins)
JGI20225J20221_1000017284	0	1	1	1048	C	4.2.1.-	K01681	330	Aconitase A
JGI20225J20221_100008488	0	1	1	1048	C	4.2.1.-	K01681	330	Aconitase A
JGI20225J20221_10008675	0	1	1	1905	C	1.6.5.3	K00334	1257	NADH:ubiquinone oxidoreductase 24 kD subunit
JGI20225J20221_10000011473	0	1	1	1142	C		K15827	13247	Fe-S-cluster-containing hydrogenase components 2
JGI20225J20221_1000014888	0	1	1	527	E	2.7.2.4	K00928	696	Aspartokinases
JGI20225J20221_1000011669	0	1	1	498	E	4.2.3.1	K01733	291	Threonine synthase
JGI20225J20221_1000015224	0	1	1	119	E	2.3.3.13	K01649	682	Isopropylmalate/homocitrate/citramalate synthases
JGI20225J20221_10068162	0	1	1	65	E	4.2.1.33	K01703	330	3-isopropylmalate dehydratase large subunit
JGI20225J20221_10000012204	0	1	1	339	E	3.4.24.70	K01414	1432	Zn-dependent oligopeptidases
JGI20225J20221_1000026377	0	1	1	112	E	2.1.2.1	K00600	464	Glycine/serine hydroxymethyltransferase
JGI20225J20221_10001658	0	1	1	19	E	4.1.1.20	K01586	2784	Diaminopimelate decarboxylase
JGI20225J20221_100035073	0	1	1	548	E	2.7.2.8	K00930	696	Acetylglutamate kinase
JGI20225J20221_1000026274	0	1	1	458	EF	6.3.5.5	K01955	2786	Carbamoylphosphate synthase large subunit (split gene in MJ)
JGI20225J20221_1000029122	0	1	1	28	EH	2.2.1.6	K01652	2776	Thiamine pyrophosphate-requiring enzymes [acetolactate synthase, pyruvate dehydrogenase (cytochrome), glyoxylate carboliga
JGI20225J20221_100067716	0	1	1	329	EM	4.2.1.52	K01714	701	Dihydrodipicolinate synthase/N-acetylneuraminate lyase
JGI20225J20221_1000079423	2	3	1	493	ER	1.4.1.13	K00266	14691	NADPH-dependent glutamate synthase beta chain and related oxidoreductases
JGI20225J20221_1000018588	0	1	1	493	ER	1.4.1.13	K00266	14691	NADPH-dependent glutamate synthase beta chain and related oxidoreductases
JGI20225J20221_100247513	0	1	1	3185	ER	4.4.1.5	K01759	13669	
JGI20225J20221_100054345	1	2	1	138	F	2.1.2.3	K00602	1808	AICAR transformylase/IMP cyclohydrolase PurH (only IMP cyclohydrolase domain in Aful)
JGI20225J20221_1000026170	0	1	1	152	F	6.3.2.6	K01923	1259	Phosphoribosylaminoimidazolesuccinocarboxamide (SAICAR) synthase
JGI20225J20221_1000014332	0	1	1	34	F	2.4.2.14	K00764	310	Glutamine phosphoribosylpyrophosphate amidotransferase
JGI20225J20221_100018681	0	1	1	47	F	6.3.5.3	K01952	13507	Phosphoribosylformylglycinamidine (FGAM) synthase, glutamine amidotransferase domain
JGI20225J20221_10014822	0	1	1	104	F	6.3.4.4	K01939	709	Adenylosuccinate synthase
JGI20225J20221_1000038303	3	4	1	191	G	4.1.2.13	K01624	1116	Fructose/tagatose bisphosphate aldolase

Locus tag	F18 (inactive)*	F19 (active)*	F19-F18 diff.*	COG	COG alpha	EC	KO	Pfam	Best JGI annotation
JGI20225J20221_100066629	1	2	1	574	G	2.7.9.1	K01006	2896	Phosphoenolpyruvate synthase/pyruvate phosphate dikinase
JGI20225J20221_1000194134	0	1	1	57	G	1.2.1.12	K00134	2800	Glyceraldehyde-3-phosphate dehydrogenase/erythrose-4-phosphate dehydrogenase
JGI20225J20221_1000079279	0	1	1	363	G	3.5.99.6	K02564	1182	6-phosphogluconolactonase/Glucosamine-6-phosphate isomerase/deaminase
JGI20225J20221_1000014367	0	1	1	837	G	2.7.1.2	K00845	2685	Glucokinase
JGI20225J20221_1000001284	0	1	1	676	G	5.1.3.15	K01792	1263	Uncharacterized enzymes related to aldose 1-epimerase
JGI20225J20221_1000038317	0	1	1	3345	G	3.2.1.22	K07407	2065	Alpha-galactosidase
JGI20225J20221_1000064217	0	1	1	4284	G	2.7.7.23	K00972	1704	UDP-glucose pyrophosphorylase
JGI20225J20221_10258832	0	1	1	448	G	2.7.7.27	K00975	483	ADP-glucose pyrophosphorylase
JGI20225J20221_10000011430	3	4	1	2918	H	6.3.2.2	K01919	4262	Gamma-glutamylcysteine synthetase
JGI20225J20221_100042437	0	1	1	2138	H	4.99.1.3	K03795	1903	Uncharacterized conserved protein
JGI20225J20221_10000041350	0	1	1	111	HR	1.1.1.95	K00058	2826	Phosphoglycerate dehydrogenase and related dehydrogenases
JGI20225J20221_100002650	0	1	1	183	I	2.3.1.9	K00626	108	Acetyl-CoA acetyltransferase
JGI20225J20221_100219016	0	1	1	236	IQ	6.1.1.13	K14188	550	Acyl carrier protein
JGI20225J20221_10000011010	0	1	1	1028	IQR	1.1.1.303	K03366	106	Dehydrogenases with different specificities (related to short-chain alcohol dehydrogenases)
JGI20225J20221_10118631	0	1	1	480	J		K02355	3764	Translation elongation factors (GTPases)
JGI20225J20221_100069279	3	4	1	724	J			76	RNA-binding proteins (RRM domain)
JGI20225J20221_100051636	3	4	1	724	J			76	RNA-binding proteins (RRM domain)
JGI20225J20221_1000540123	4	5	1	724	J			76	RNA-binding proteins (RRM domain)
JGI20225J20221_100148219	2	3	1	480	J		K02355	3764	Translation elongation factors (GTPases)
JGI20225J20221_1000250153	0	1	1	1185	J	2.7.7.8	K00962	1138	Polyribonucleotide nucleotidyltransferase (polynucleotide phosphorylase)
JGI20225J20221_100002931	0	1	1	233	J		K02838	1765	Ribosome recycling factor
JGI20225J20221_100031374	0	1	1	96	J		K02994	410	Ribosomal protein S8
JGI20225J20221_100033719	0	1	1	13	J	6.1.1.7	K01872	1411	Alanyl-tRNA synthetase
JGI20225J20221_10000041375	0	1	1	2092	J		K03232	736	Translation elongation factor EF-1beta
JGI20225J20221_1000014181	0	1	1	143	J	6.1.1.10	K01874	9334	Methionyl-tRNA synthetase
JGI20225J20221_1000026340	0	1	1	215	J	6.1.1.16	K01883	1406	Cysteinyl-tRNA synthetase
JGI20225J20221_1000004386	0	1	1	2058	J		K02869	428	Ribosomal protein L12E/L44/L45/RPP1/RPP2
JGI20225J20221_1000011689	0	1	1	2004	J		K02974	1282	Ribosomal protein S24E
JGI20225J20221_1000045536	0	1	1	99	J			416	Ribosomal protein S13
JGI20225J20221_1000050102	0	1	1	50	J		K02358	9	GTPases - translation elongation factors
JGI20225J20221_100068361	0	1	1	72	J	6.1.1.20	K01890	3483	Phenylalanyl-tRNA synthetase beta subunit
JGI20225J20221_10540811	0	1	1	480	J		K02355	3764	Translation elongation factors (GTPases)
JGI20225J20221_1000015552	1	2	1	1846	K			13463	
JGI20225J20221_100031387	0	1	1	202	K	2.7.7.6	K03040	3118	DNA-directed RNA polymerase, alpha subunit/40 kD subunit
JGI20225J20221_1000079759	0	1	1	217	KJ			1709	Uncharacterized conserved protein - COG0217
JGI20225J20221_1000014643	1	2	1	449	M	2.6.1.16	K00820	1380	Glucosamine 6-phosphate synthetase, contains amidotransferase and phosphosugar isomerase domains
JGI20225J20221_1000004282	0	1	1	84	N	3.1.21.-	K03424	1026	Mg-dependent DNase
JGI20225J20221_1000084432	7	8	1	443	O		K04043	12	Molecular chaperone
JGI20225J20221_10029619	7	8	1	443	O		K04043	12	Molecular chaperone
JGI20225J20221_10000041040	0	1	1	443	O		K04043	12	Molecular chaperone
JGI20225J20221_100042718	0	1	1	1730	O		K04797	2996	Predicted prefoldin, molecular chaperone implicated in de novo protein folding - COG1730
JGI20225J20221_100151118	0	1	1	1123	O		K00400	5	ATPase components of various ABC-type transport systems, contain duplicated ATPase
JGI20225J20221_10000012049	0	1	1	1047	O	5.2.1.8	K03775	254	FKBP-type peptidyl-prolyl cis-trans isomerases 2
JGI20225J20221_1000029145	0	1	1	760	O	5.2.1.8	K03771	9312	Parvulin-like peptidyl-prolyl isomerase
JGI20225J20221_100002945	0	1	1	316	O		K15724	1521	Uncharacterized conserved protein - COG0316
JGI20225J20221_1000079657	0	1	1	822	O			1592	NifU homolog involved in Fe-S cluster formation
JGI20225J20221_100015641	0	1	1	544	O		K03545	5697	FKBP-type peptidyl-prolyl cis-trans isomerase (trigger factor)
JGI20225J20221_100146625	0	1	1	2998	P		K05772	12849	ABC-type tungstate transport system, permease component
JGI20225J20221_100078020	0	1	1	667	R			248	Predicted oxidoreductases (related to aryl-alcohol dehydrogenases) - COG0667
JGI20225J20221_1000011565	0	1	1	535	R		K02585	4055	Radical SAM superfamily
JGI20225J20221_100042426	0	1	1	1710	S			8004	Uncharacterized protein conserved in archaea
JGI20225J20221_10086442	0	1	1						
JGI20225J20221_10291582	0	1	1						
JGI20225J20221_100018699	0	1	1						
